# Variation of growth and transcriptome responses to arbuscular mycorrhizal symbiosis in different foxtail millet lines

**DOI:** 10.1186/s40529-023-00391-y

**Published:** 2023-06-16

**Authors:** Ou-Chi Chang, Wei-Yi Lin

**Affiliations:** Department of Agronomy, National Taiwan University, Taipei, 106319 Taiwan

**Keywords:** Arbuscular mycorrhizal fungi, Foxtail millet, Symbiosis, Transcriptome

## Abstract

**Background:**

Arbuscular mycorrhizal fungi (AMF) have been applied to promote the growth of different crop species, but knowledge about the impacts of symbiosis on foxtail millet at the physiological and molecular levels have remained limited. In this study, we compared the mycorrhization phenotypes of one cultivar and three different landraces and performed a comprehensive transcriptomic analysis to assess the effects of genetic variation on the responses to symbiosis.

**Results:**

Our results showed that colonization by AMF did not enhance biomass accumulation but significantly increased grain production only in three lines. More than 2,000 genes were affected by AMF colonization in all lines. Most AM symbiosis-conserved genes were induced, but the induction levels varied between lines. Gene Ontology (GO) analysis showed that Biological Function terms related to nitrogen transport and assimilation were only enriched in TT8. Similarly, two of phosphate starvation-induced phosphate transporters were only simultaneously downregulated in TT8. In the other two lines, the enrichment of GO terms associated with cell wall reorganization and lignification was observed, though the effects were different.

**Conclusion:**

This study reveals the impacts of genetic variation of millet lines on the responses to AM symbiosis and provides information regarding AMF application for millet production.

**Supplementary Information:**

The online version contains supplementary material available at 10.1186/s40529-023-00391-y.

## Introduction

Arbuscular mycorrhizal fungi (AMF) are soil-born fungi that belong to the phylum Glomeromycota. They are obligate endosymbionts and can form beneficial mutualism with more than 80% of land plant species (Smith and Read 2008). AMF acquire mineral nutrients through extensive hyphal networks and transfer to host plants in exchange for essential carbon sources through the interface between arbuscule membrane and periarbuscular membrane, the plant cell membrane encircled arbuscule (Roth and Paszkowski 2017). Thus, the formation of AM symbiosis (AMS) enhances the nutrient uptake in host plants and the survival rate under poor nutrient conditions (Smith and Smith 2011). In addition, AMS can also improve the abiotic and biotic stress resistance of host plants (Dowarah et al. 2021; Lenoir et al. 2016).

Based on fossil evidence, the association of AMF and plants developed in the early stage of land plant evolution (Heckman et al. 2001; Taylor et al. 1995). Common symbiotic processes including molecular regulatory mechanisms and cell development are controlled by a set of genes that are highly conserved in AM host plants (Bravo et al. 2016; Delaux et al. 2014). For example, *STR*, encoding an ABC transporter, and *DMI2*, encoding a receptor-like kinase, are essential genes for AMS and only present in AM plant species (Endre et al. 2002; Zhang et al. 2010).

The genome-wide analysis of AMS-responsive genes has been reported for many host plant species, such as *Medicago truncatula*, *Solanum lycopersicum*, *Helianthus annuus*, *Oryza sativa*, and *Triticum aestivum* (Fiorilli et al. 2009; Gutjahr et al. 2015; Li et al. 2018; Liu et al. 2003; Vangelisti et al. 2018), identifying a core set of genes involved in AMS. However, the comparative transcriptomic analysis of conserved AMS-responsive genes in different host plant species has revealed species-specific expression patterns, suggesting the effects of the genetic structure of host plants on the molecular regulation of AMS (An et al. 2018). Mateus et al. (2019) and Watts-Williams et al. (2019) further pointed out strong impacts of the genetic variation between genotypes on the interaction between hosts and AMF.

Foxtail millet (*Setaria italica* (L.) P. Beauv) is an ancient crop that was domesticated around 6,000 B.C. in China (Austin 2006). The grains are rich in starch, protein and fibers (Saleh et al. 2013), and they are the staple foods in semi-arid regions of Asia and as fodder in Europe, North America, Australia, and North Africa. Due to its short growth season and adaptation to abiotic stresses such as drought stress, foxtail millet has become a nutritious crop that has significant potential to meet nutrition demands during climate change (Sachdev et al. 2021). Moreover, the genome sequence has been released (Bennetzen et al. 2012; Li and Brutnell 2011; Zhang et al. 2012), so it is considered a model plant for studying stress resilience. However, we know little about the impacts of AMS on the morphology, physiology and molecular aspects of this species. Ceasar et al. (2014) first demonstrated the expression of members of phosphate transporter family 1 (PHT1) in mock and AMF-inoculated foxtail millets and identified SiPHT1;8 and SiPHT1;9 as AMS-responsive phosphate transporters that belong to monocotyledon- and AM host-specific lineages, respectively. They also reported an increase in seed weight in AMF-treated plants. However, details regarding the influence of AMS on foxtail millets are still required to understand the efficiency of AMF application in the field.

In Taiwan, foxtail millets are widely used by indigenous people, and more than 160 landraces with great genetic diversity have been recorded (Lin et al. 2012). For example, among 124 landraces collected in Taiwan, four *Waxy* alleles have been identified, resulting in different amylose content in the grains (Kuo et al. 2018). To gain insights into the impacts of AMS on foxtail millet and the effects of genetic variation on the benefit of AMS, we investigated the growth and yield of one cultivar and three different foxtail millet landraces and analyzed AMS-responsive genes through RNA-sequencing technology. Although symbiosis did not benefit the growth of millets, the yield of at least two lines was significantly increased. Transcriptomic analyses showed the differential responses to AMS at molecular levels in three millet lines. Our studies revealed the contribution of the intraspecific genetic variation of host plants to symbiotic responses at the physiological and molecular levels, which needs to be considered for AMF application in the future.

## Materials and methods

### Plant growth conditions and AMF inoculation

One Taiwan cultivar TT8 and three different foxtail millet landraces (Hanevalval, ISE36 and ISE42) were used in this study. Seeds were surface-sterilized and germinated in a growth chamber with a 12 h light (28 °C)/ 12 h dark (22 °C) cycle. At two weeks post germination, plants were transplanted into sterile cones or 6-inch pots filled with autoclaved sand and peat moss (Blumen Erde, Euflor, Germany) mixed at an 8:2 ratio and inoculated with 10 mL of *Claroideoglomus etunicatum* inoculants containing around 1000 spores. Plants were supplied with 7 and 50 ml liquid fertilizer containing N: P_2_O_5_: K_2_O = 15: 5: 25 per cone and pot every two weeks, respectively. For the transcriptome analysis, plants were harvested at six weeks post transplanting. The fresh weight and length of shoots and roots were measured. The leaves were harvested for phosphate and anthocyanin concentration analysis. The roots were cut in half; one part was frozen for RNA extraction and the other part was used for AMF staining. For the yield investigation, plants were kept growing in six-inch pots until seed maturation. The length and dry weight of panicles, number of seeds per panicle, and thousand seed weight were recorded.

### Measurement of inorganic phosphate and anthocyanin concentration

Phosphate concentration was measured based on the modified method described by Chiou et al. (2006). Briefly, leaves were homogenized with an extraction buffer (1% acetic acid) at a ratio of 1 mg of sample to 10 µL of buffer. The homogenates were incubated at 42 °C for 30 min and then centrifuged at 13,000 rpm for 10 min. The supernatant was mixed with an assay solution (0.35% NH_4_MoO_4_, 0.86 N H_2_SO_4_ and 1.4% ascorbic acid) and incubated at 42 °C for 30 min before phosphate content measurement at A_820_ using spectrometry (Molecular Devices, USA).

For anthocyanin concentration analysis, the method described by Lange et al. (1971) was followed. Leaves were ground into powder and a 1 mL extraction buffer containing 1-propanol: HCl: QH_2_O = 18: 1: 81 was added. After mixing by vortexing, samples were left at 100 °C for 90 s and placed on ice to stop the reaction. The tubes were subjected to high-speed centrifugation at 4 °C and the supernatant was used for measuring anthocyanin content at A_535_ and A_650_ using spectrometry (Molecular Devices). The concentration was expressed in the following equation.

$$\begin{aligned}& Anthocyanin\,concentration\, \\ & \quad = \,\left( {{A_{535}}\, - \,2\, \times \,{A_{650}}} \right)\,/\,fresh\,weight.\end{aligned}$$

### AMF staining and quantification of colonization

The percentage of AMF colonization in roots was estimated after trypan blue staining using a method described previously (Phillips and Hayman 1970). Briefly, roots were fragmented and immersed in 10% (w/v) KOH at 90 °C for 30 min for clearing. After several washings with tap water, roots were acidified by 0.3 N HCl for 30 min. Then the roots were stained using trypan blue (0.1% w/v dissolved in a solution of 50% lactic acid, 25% glycerol and 25% ddH_2_O) overnight. Next, the roots were de-stained using acidic glycerol (glycerol: 0.3 N HCl = 1: 1) and stored at 4 °C.

Colonization was assessed based on the gridline intersect method described by McGonigle et al. (1990) and expressed as the percentage of colonized root length (the count of colonized root intersections/ the total number of root intersections). At least 100 roots fragments were randomly selected for evaluation and visualization with an Olympus SZX-16 stereomicroscope (Olympus, Japan).

### Total RNA isolation and RNA sequencing

Around 100 mg of root tissues was ground in pre-chilled mortars and pestles in liquid nitrogen and total RNA was isolated using the method described by Wang and Vodkin (1994). The RNA was dissolved in nuclease-free water and stored at -80 °C before further processing. Four replicates were prepared for each treatment.

The quantity and purity of RNA were evaluated using a SimpliNano™ spectrophotometer (Biochrom, USA) and the integrity of RNA was assessed using Qsep 100 DNA/ RNA Analyzer (BIOptic, Taiwan). A total amount of 1 µg RNA per sample was used for RNA sequencing. The libraries were generated using a KAPA mRNA HyperPrep Kit (Roche, Switzerland) and 300–400 bp fragments containing adaptors were sorted using a KAPA Pure Beads system (Roche) following the manufacture’s instruction. Sequencing was performed on a NovaSeq 6000 platform (Illumina, USA).

### RNA sequencing analysis

Clean reads were extracted by removing low quality reads and trimming adapter sequences using Trimmomatic v0.38 (Bolger et al. 2014) and were aligned to *Setaria italica* genome v2.0 (Bennetzen et al. 2012) using HISAT2 v2.1.0 (Kim et al. 2015; Sahraeian et al. 2017). The read numbers mapped to the individual genes were counted by featureCounts (v2.0) (Liao et al. 2014). For relative gene expression analysis, normalization and differential expression gene (DEG) analysis were performed using edgeR (v3.28.1) and DESeq2 (v.1.26.0), respectively (Anders et al. 2013; Li et al. 2016; Love et al. 2014). The *p*-values were adjusted using Benjamini and Hochberg’s approach for controlling the false discovery rate (FDR). The thresholds of DEGs were set as FDR ≤ 0.05 and absolute log2 fold change ≥ 1.

The Gene Ontology (GO) enrichment analyses of DEGs was carried out using clusterProfiler v3.14.3 with a corrected FDR < 0.05 (Yu et al. 2012).

### Statistic analysis

Data were analyzed with a two-way ANOVA followed by Tukey’s Honest Significant Difference test to evaluate the statistical significance of the difference between samples.

## Results

### The effects of AMS on the growth and phosphate accumulation of different foxtail millet lines

The effects of genetic variation on the responses to environmental phosphate content have been well-demonstrated in many species, including foxtail millet (Ceasar et al. 2020). To understand the variations of AMS responses in different genotypes, we first evaluated the morphological and physiological responses of different millet lines including eleven landraces and one Taiwan cultivar to AMS and found differential symbiotic phenotypes and variation of AMF colonization efficiency (Additional file 1: Fig. S1). Hanevalval and TT8, a landrace and a cultivar from Taiwan, respectively, and the other two landraces from India, ISE36 and ISE42 (which had similar AMF colonization efficiencies but showed different responses to AMS), were selected for further investigation. At six weeks post inoculation, plants were harvested for physiological analysis (Additional file 1: Fig. S2). The colonization efficiency of AMF in these four lines was similar (Additional file 1: Fig. S3a). However, different from the previously reported benefits of AMS to plant growth (Begum et al. 2019), the shoot lengths of Hanevalval and ISE36 were not affected by AMS but those of AMF-colonized TT8 and ISE42 were even shorter than mock-treated plants (Additional file 1: Figs. S2 and S3b). For root length, there was no difference between mock- and AMF-treated plants (Additional file 1: Fig. S3c). Similarly, the shoot and root fresh weight of Hanevalval was not affected by AMF, but AMF-colonized TT8, ISE36 and ISE42 were significantly reduced compared with mock-treated ones (Additional file 1: Figs. S3d and e).

Although the growth of these lines was not enhanced by AMS, the shoot phosphate concentration was significantly increased in all lines that were inoculated by AMF (Additional file 1: Fig. S3f), suggesting that AMS was able to promote phosphate accumulation in host plants but did not directly benefit the growth of foxtail millet. Anthocyanin accumulation is one of characteristics of phosphate starvation responses (Raghothama 2000). The leaves of TT8 became purple by low phosphate treatments, while the leaves of other three landraces remained green under low phosphate conditions. AMF inoculation did not affect anthocyanin accumulation in TT8 (Additional file 1: Fig. S2). The quantitative measurement of anthocyanin content coincided with the observation (Additional file 1: Fig. S3g), suggesting that low phosphate-induced anthocyanin accumulation in shoots is determined by genotype and AMF colonization had no or minor effects on this phenotype.

### The effects of AMS on the yield of different foxtial millet lines

**Fig. 1:**
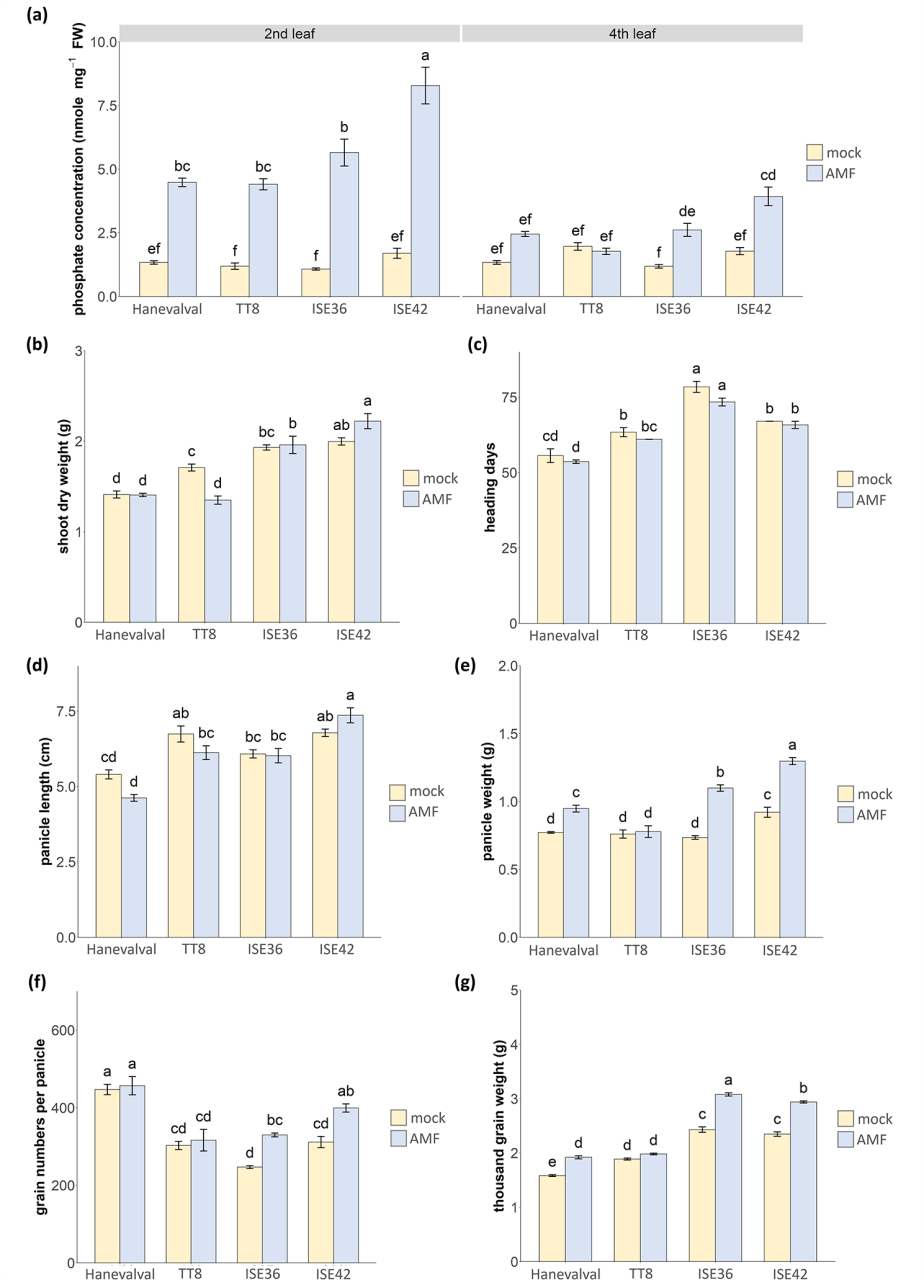
The effects of AMS on the phosphate accumulaton and yields of four different foxtail millet lines. **a**, Shoot phosphate concentration in second and fourth young leaves, respectively; **b**, Shoot dry weight; **c**, Heading date; **d**, Panicle length; **e**, Panicle weight; **f**, Grain number per panicle; **g**, Thousand grain weight. n = 5. Values are mean ± SE. Data were analyzed with ANOVA (*p* < 0.05). Different characters over bars indicate significant differences

To further understand whether the association with AMF benefits the yield and how performance varies between foxtail millets with different genetic background, the millet seedlings were transplanted to pots after inoculation. At 6 weeks post transplanting, the second and fourth young leaves were harvested to analyze phosphate concentration. Interestingly, the phosphate concentration of the second young leaves were significantly higher in all the AMF-associated plants, especially in ISE42, while in fourth young leaves, the increase in phosphate concentration by AMS was only observed in ISE36 and ISE42 (Fig. 1a). These results suggested that the distribution of excess phosphate provided by AMF might differ between lines. Similar to the growth responses to AMF shown in Additional file 1: Fig. S3, the shoot dry weight of AMF-treated TT8 was significantly reduced compared with mock-treated plants, but Hanevalval, ISE36 and ISE42 were not affected by AMS (Fig. 1b). All the plants were grown in a greenhouse until panicles were harvested. Under pot conditions, most plants had only one panicle regardless of the difference of lines (Additional file 1: Fig. S4). Of the four lines, the heading date of Hanevalval was earliest, followed by those of TT8 and ISE42, and the vegetative growth period of ISE36 was the longest, but the heading date was not affected by fungal colonization (Fig. 1c). We measured the panicle length, panicle weight, grain number per panicle and thousand grain weight. The panicle length was the shortest in Hanevalval and was the longest in ISE42 (Fig. 1d). Although the panicle length was not affected by AMS, the panicle weight was significantly increased in AMF-treated Hanevalval, ISE36 and ISE42 (Fig. 1e). The increase in grain number per panicle by AMF was only observed in two Indian landraces (Fig. 1f), but the thousand grain weight in Hanevalval, ISE36 and ISE42 was increased by symbiosis (Fig. 1g). In summary, benefits of AMS on foxtail millet production were observed, but the effects varied between lines.

### Transcriptomic analysis of different foxtail millet lines during AMS

**Fig. 2:**
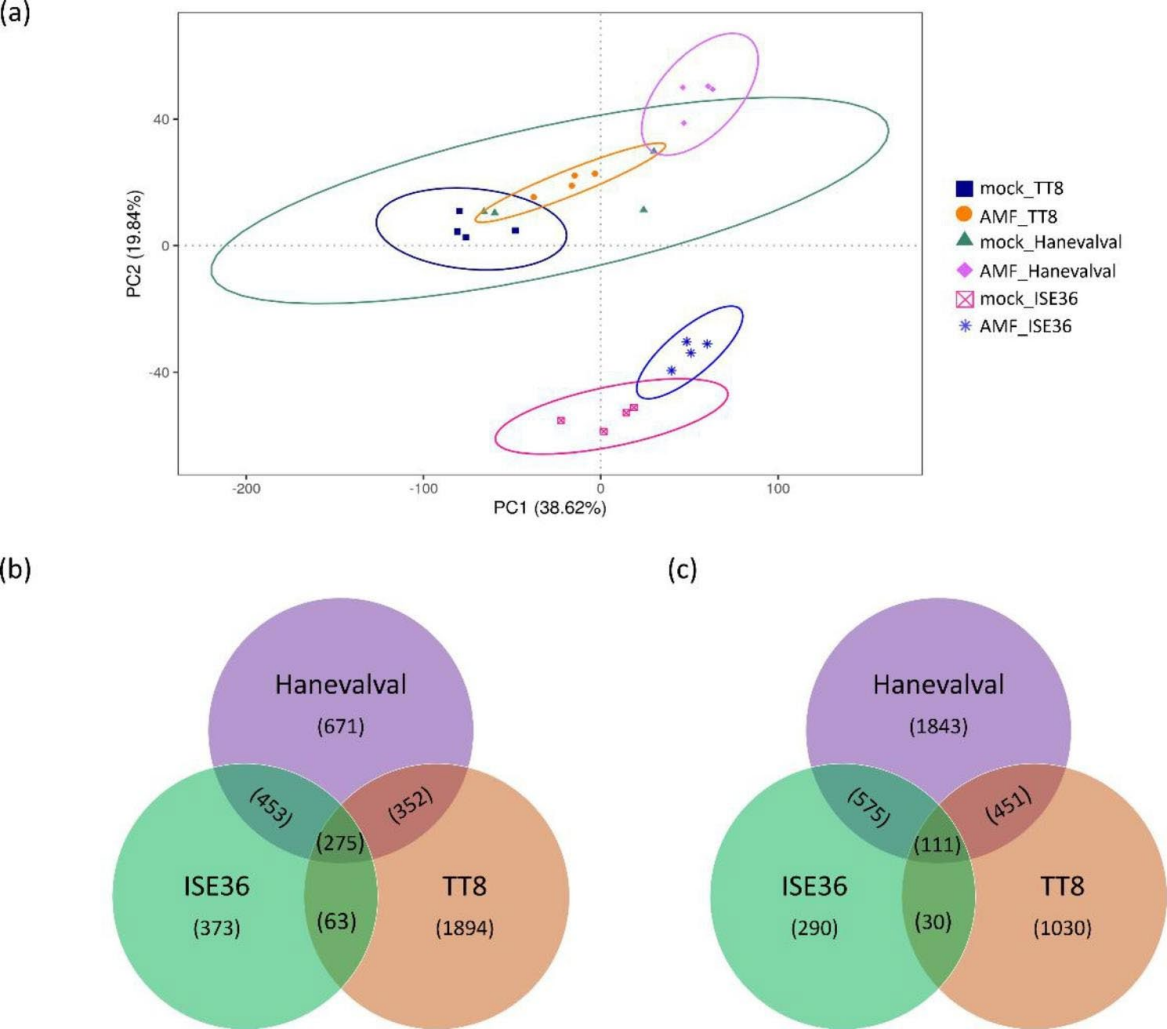
The overview of transcriptomic data derived from AMF- and mock-treated roots of different millet lines. **a**, PCA plot with 95% confidence ellipses; **b** and **c**, Venn diagrams showing the number of up- and downregulated genes, respectively, in three lines in response to AMS

In order to understand the effects of genetic variance on the AMS responses at the molecular level in the roots of foxtail millet, we performed the transcriptome analysis of mock- and AMF-treated roots of Hanevalval, TT8 and ISE36. After trimming and filtering, the number of high-quality reads of each library ranged from 40,226,358 to 55,208,702 and the GC content was around 52–53% (Additional file 1: Table S1). The percentage of high-quality reads mapped to the reference genome ranged from 87.1 to 96.1%, and less than 5% of reads were multi-mapped (Additional file 1: Table S2). Principle component analysis was performed to evaluate the similarity of samples within the same group, and the results showed that four biological samples of the same group were well-clustered except for mock-treated Hanevalval. Moreover, mock- and AMF-treated samples were clearly separated (Fig. 2a), indicating the reliability of the results from biological replicates and the differential responses to mock and AMF treatments.

The expression level of more than 80% of DEGs was changed 2-to-8 fold by AMS. In Hanevalval, 4,731 DEGs with a fold change of ≧ 2 and adjusted *p*-value of < 0.05 were identified in response to AMS, including 1,751 and 2980 up- and downregulated genes, respectively. In TT8, we found a total number of 4,206 DEGs, including 2,584 and 1,622 genes induced and repressed in AMF-treated roots, respectively. In ISE36, we identified 2,170 DEGs, including 1,164 and 1,006 up- and downregulated genes, respectively. Comparing all the DEGs revealed that only 275 and 111 up- and downregulated genes, respectively, were commonly present in these three different genetic backgrounds. More than 60% of upregulated DEGs were specifically identified in TT8; around 38% and 61% of up- and downregulated DEGs were Hanevalval-specific; and less than 35% DEGs specifically responded in ISE36 (Fig. 2b and c), implying the specificity of molecular responses to AMS in these lines.

### Differential expression of AMS-conserved genes in different millet lines

Genes that are essential in symbiotic signaling pathways and arbuscule development are usually conserved across host plant species. Through phylogenomic analysis, more than one hundred genes have been identified as AMS-conserved genes (Bravo et al. 2016; Delaux et al. 2014; Favre et al. 2014). We investigated the expression of orthologs of AMS-conserved genes reported by Bravo et al. (2016) in three lines to assess the conservation of these gene lineages in foxtail millet. Among 54 orthologs found in foxtail millet, most of them were induced by AMS. However, only 19 genes were commonly affected by AMS in all three lines, including *SiPHT1;9* and the orthologs of *RAM1*, *RAM2*, *STR2*, *FatM*, and *VAPYRIN*, which are well-studied AMS-conserved genes (Bravo et al. 2017; Ceasar et al. 2014; Gobbato et al. 2013; Murray et al. 2011; Park et al. 2015). The relative expression levels of other AMS-conserved genes varied between lines. For example, the orthologs of *RAD1* and a gene encoding a DnaJ domain protein were only induced by AMS in TT8, and the ortholog of *AMT2;1* was only induced in Hanevalval and TT8. In addition, only eleven genes were exclusively AMS-induced genes (Table S3), while most AMS-conserved genes in *Medicago* specifically responded to AMS (Bravo et al. 2016).

We also compared the expression levels of genes belong to the core set of AMS-responsive genes which show similar symbiotic responses in at least two other plant species (An et al. 2018). Among 116 ortholog groups in the core set of AMS-responsive genes, the number of ortholog groups which showed similar expression pattern was highest in TT8 (71), and lowest in ISE36 (35) (Additional file: Fig. S5), supporting the conservation of the core transcriptional program in three millet lines but with different induction levels. Taken together, our results showed that the difference of genetic variation between millet lines affected the expression level of AMS-responsive genes.

### The impacts of AMS on gene expression profiles of foxtail millet

**Fig. 3:**
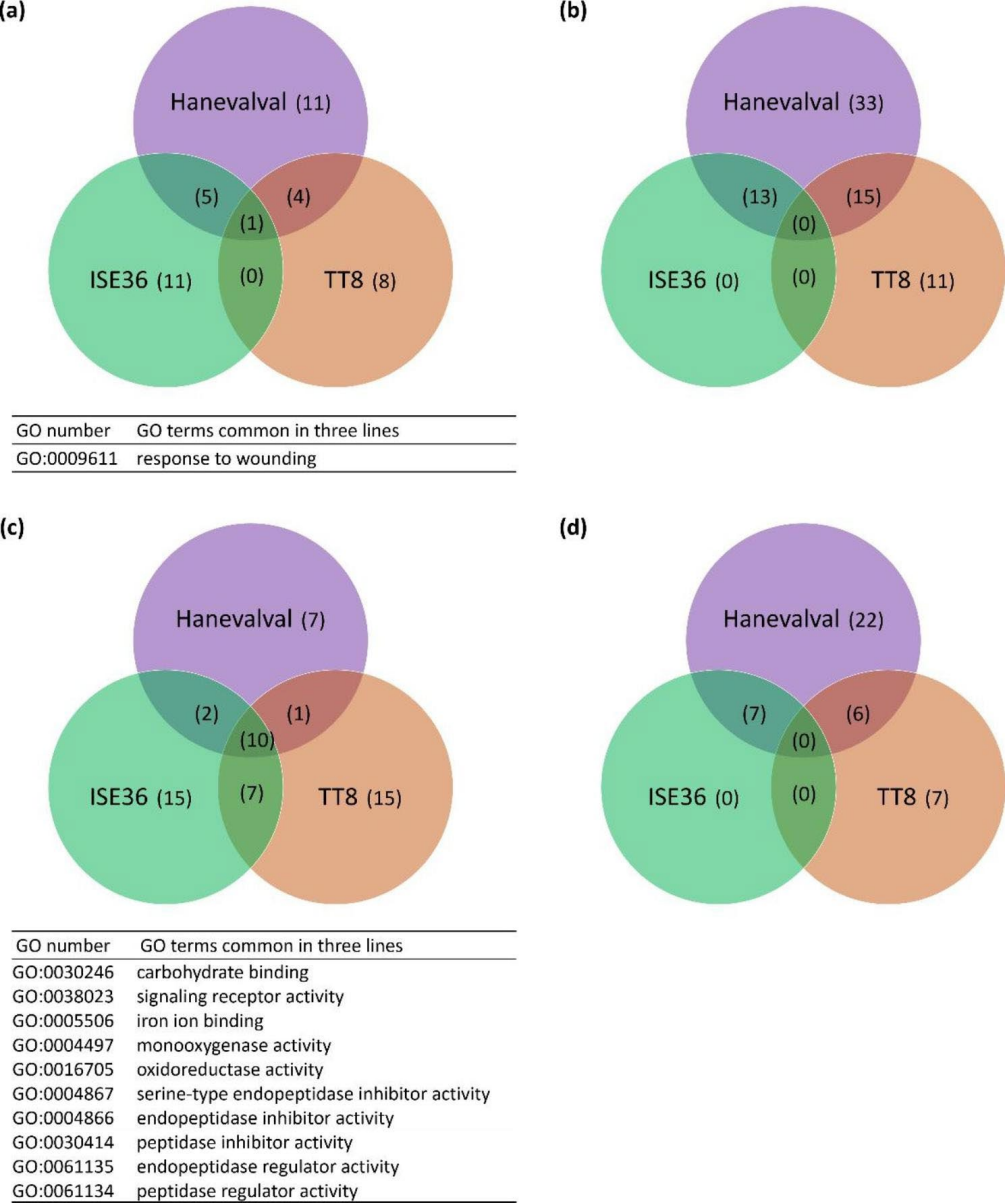
Venn digarms of GO terms significantly enriched in the three different lines. **a** and **b**, Enriched Bological Process terms in up- and downregulated DEGs, respectively; **c** and **d**, Enriched Molecular Function terms in up- and downregulated DEGs, respectively. Adjusted *p*-value < 0.05. The GO terms commonly enriched in all three lines were listed below the diagrams

To further elucidate the genome-wide effects of genetic variance on AMS responses, GO analysis was performed to dissect the functions of DEGs. Among Biological Process classes, there were 21, 11 and 17 terms were significantly enriched in upregulated genes in Hanevalval, TT8 and ISE36 but only one term (GO: 0009611 response to wounding) is commonly present in all three lines (Fig. 3a). Six terms associated with amino acid metabolisms were specifically identified in Hanevalval and two associated with nitrate responses were enriched only in TT8 (Additional file: Table S4). In ISE36, terms associated with lignin metabolic processes were dominant in the upregulated DEGs (Additional file: Table S4 and Fig. S6a). Regarding Molecular Function terms enriched in the upregulated gene profiles, ten terms were commonly present in all three lines, and the four in top ten list were “iron ion binding (GO: 0005506)”, “monooxygenase activity (GO: 0004497)”, “carbohydrate binding (GO: 00030246)”, and “serine-type endopeptide inhibitor activity (GO: 0004867)” (Fig. 3c and Additional files: Fig. S6b). In TT8, among 15 terms specifically enriched in upregulated DEGs, 8 were associated with transmembrane transporters and substrate movement. Among the terms specifically enriched in ISE36, several terms related to enzymes involved in protein and carbohydrate degradation were dominant in the list (Additional files: Table S5).

For downregulated DEGs, there were more Biological Process terms enriched in all three lines, surprisingly, no common terms were identified (Fig. 3b). Several cell wall biogenesis and metabolsims-related terms were dominant in Hanevalval, while in TT8, Biological Process terms associated with responses to abiotic stress and ion homeostasis were enriched (Additional file: Table S4 and Fig. S7). Regarding the Molecular Function class, no common terms were enriched in all three lines (Fig. 3d). Among the terms specifically enriched in Hanevalval, “cytoskeleton synthesis and binding”-related functions were dominant, whereas in TT8, several terms associated with substrate transport were found, which echoed to the enrichment list of the Biological Process class. Different from Hanevalval and TT8, there were no Biological Process and Molecular Function terms specfically enriched in ISE36 (Fig. 3b and d). The results of GO analysis showed that the impacts of AMS on molecular responses were greatly determined by the difference of genotypes.

### Comparing the responses of nutrient transport and metabolism to AMS in three millet lines

**Fig. 4:**
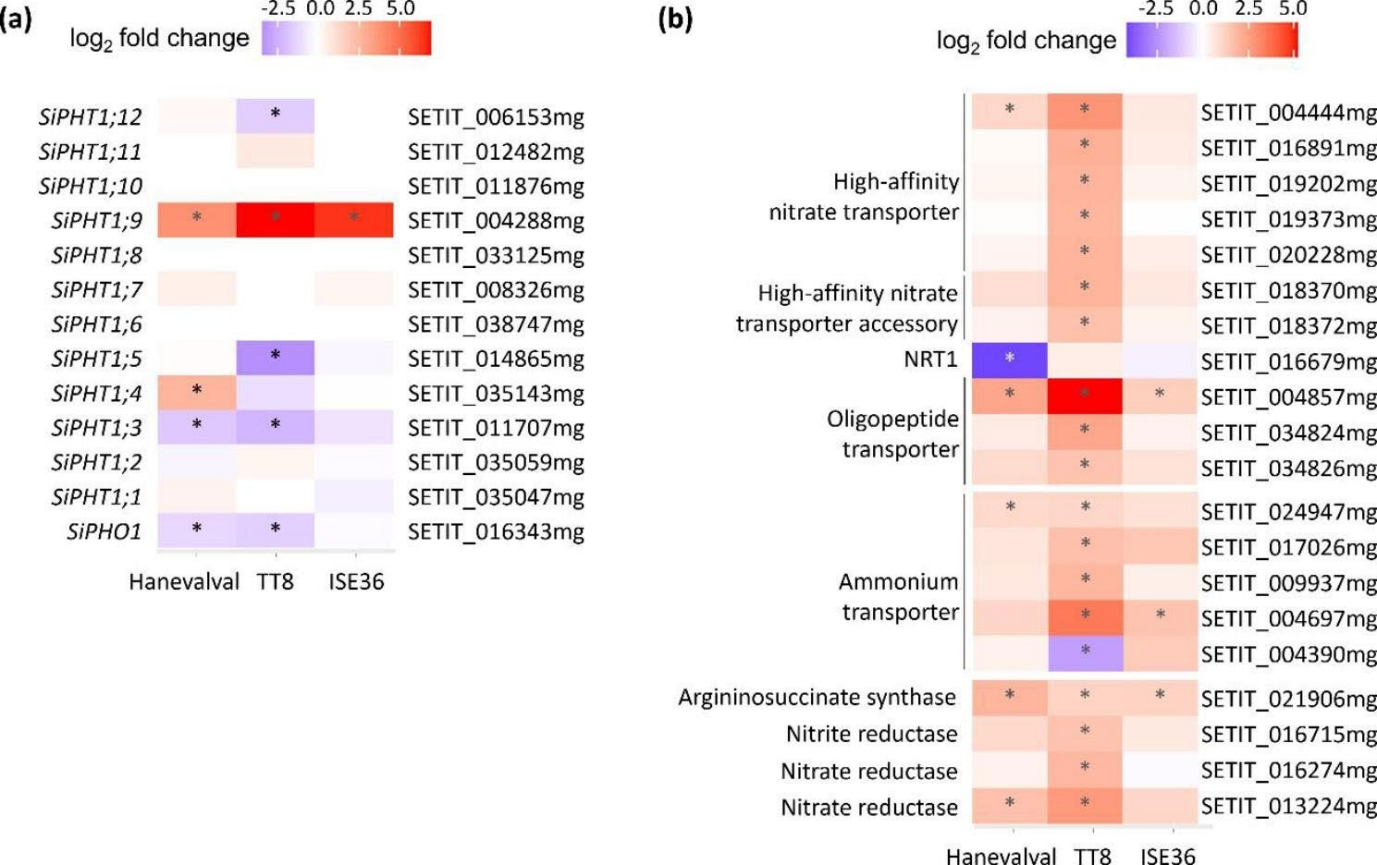
Heatmap of a, *PHT1* genes and b, gene subsets associated with nitrogen transport and assimilation. Red and purple indicate up- and downregulated genes, respectively, in AMS. *, adjusted *p*-value < 0.05

The enhancement of phosphate accumulation in host plants is one of the most well-described benefits of AMS (Smith et al. 2011; Smith and Smith 2011). In the foxtail millet genome, twelve genes were annotated as *PHT1* family members. Phylogenetic analysis showed that SiPHT1;9 was in the AMS-inducible clade conserved in both monocot and dicot host species, while SiPHT1;8 was in the monocot-specific AMS-inducible clade. Both genes were strongly induced in AMF-colonized roots in a previous study (Ceasar et al. 2014). However, we only found that *SiPHT1;9* was upregulated in mycorrhizal roots, and the induction level was much stronger in TT8 and ISE36 than in Hanevalval, while *SiPHT1;8* transcript was not detected in all three lines (Fig. 4a). We also examined and compared the transcript levels of other ten *PHT1* family members in the transcriptome profiles of the three lines. Interestingly, *SiPHT1;3*, *SiPHT1;5* and *SiPHT1;12* were significantly downregulated in TT8, while only *SiPHT1;3* was downregulated and *SiPHT1;4* was even upregulated in mycorrhizal Hanevalval roots. In ISE36, no *PHT1* members except *SiPHT1;9* were affected by AMS (Fig. 4a). It was shown that both *SiPHT1;3* and *SiPHT1;4* were induced in roots following by low phosphate treatment but did not respond to AMS (Ceasar et al. 2014). Further studies will be required to elucidate whether the differential responses of *PHT1* genes to AMS are due to the variation of internal phosphate levels.

AMF is also known to promote nitrogen uptake and metabolism (Jin et al. 2005; Thirkell et al. 2016, 2019) and the GO terms associated with nitrogen metabolism are usually enriched in DEGs (Li et al. 2018). But in this study the GO terms “nitrate transport (GO: 0015706)”, “responses to nitrate (GO: 0010167)” and “nitrogen cycle metabolism (GO: 0071941)” were only enriched in TT8. Thus, we retrieved the genes encoding ammonium transporters, nitrate transporters, oligopeptide transporters and enzymes involved in the nitrogen cycle. In TT8 roots, AMS significantly upregulated four ammonium transporters, six nitrate transporters, two nitrate transporter accessories, and two oligopeptide transporters by two-to-eleven fold, though one of ammonium transporters was downregulated. In Hanevalval and ISE36, only one or two transporters were affected. Similarly, two nitrate reductase and nitrite reductase genes were only induced in mycorrhizal TT8 roots, while only one nitrate reductase was induced by AMF in Hanevalval (Fig. 4b). In terms of genes involved in amino acid biosynthesis and metabolic pathways, several arginine and glutamine family biosynthesis- and metabolism-related genes were upregulated by AMS, mainly in Hanevalval and TT8, though the DEG profiles were not exactly the same in these two lines (Table 1). There were also many genes involved in alpha and aromatic amino acid biosynthetic and metabolic processes upregulated in Hanevalval, but less genes were affected or even downregulated by AMS in TT8 (Table 1).

**Table 1:** The relative gene expression level of genes associated with amino acid biosynthesis and metabolism in three lines

Gene ID	Gene annotation	Log2 fold change^1^		
		Hanevalval	TT8	ISE36
Glutamine family amino acid biosynthetic and metabolic processes				
SETIT_017628mg	Glutamine synthetase	1.55 *	1.27 *	0.83
SETIT_022118mg	Glutamine synthetase	0.22	1.77 *	0.68
SETIT_036352mg	Glutamine synthetase	2.09 *	0.91	2.46 *
SETIT_021485mg	UTP–ammonia ligase	0.65	1.44 *	0.28
SETIT_000703mg	UTP–ammonia ligase	0.08	1.21 *	0.48
SETIT_017204mg	Carbamoyl phosphate synthase small chain, chlroplastic	1.46 *	1.21 *	0.75
SETIT_017936mg	Glutamine amidotransferase	0.00	2.85 *	0.00
SETIT_019548mg	GMP synthase-related	-3.34 *	1.70 *	-1.28
SETIT_020943mg	Glutamate synthase (NADH)	2.13 *	1.95 *	0.90
SETIT_036070mg	pyridoxal-5-phosphate decarboxylase	0.34	1.04 *	0.10
SETIT_017738mg	N-acetylglutamate synthase	1.73	3.06 *	1.73
SETIT_021759mg	Argininosuccinate lyase	1.59 *	1.47 *	0.86
SETIT_021906mg	Argininosuccinate synthase	2.00 *	1.18 *	1.21
SETIT_021951mg	Acetylornithine transaminase	1.37 *	1.50 *	1.09
SETIT_034795mg	Amino acid acetyltransferase	1.56 *	1.13 *	0.89
SETIT_035137mg	Glutamate N-acetyltransferase	1.81 *	0.99	0.90
SETIT_035209mg	Argininosuccinate lyase	1.42 *	0.06	0.90
SETIT_035869mg	N-acetyl-gamma-glutamyl-phosphate reductase	2.13 *	0.97	1.53 *
SETIT_035474mg	Ornithine aminotransferase	0.89	-0.21	1.03 *
SETIT_007956mg	Arginine decarboxylase	0.44	1.01 *	-0.37
SETIT_035342mg	Proline oxidase	-0.84	1.47 *	-1.05
Aromatic amino acid family metabolic process				
SETIT_001761mg	Subgroup I aminotransferase related	1.16 *	-0.09	0.77
SETIT_029865mg	3-dehydroquinate synthase	1.63 *	0.76	0.91
SETIT_035017mg	3-deoxy-7-phosphoheptulonate synthase	1.30 *	0.49	0.42
SETIT_000995mg	Shikimate dehydrogenase	1.10 *	0.19	0.47
SETIT_021701mg	Shikimate dehydrogenase	1.22 *	0.65	0.46
SETIT_017907mg	Shikimate kinase 1, chloroplatic-related	1.30 *	0.35	1.00 *
Aromatic amino acid family metabolic process				
SETIT_006296mg	3-phosphoshikimate 1-carboxyvinyltransferase	1.43 *	1.14 *	0.62
SETIT_035737mg	Chorismate synthase	1.44 *	0.73	0.71
SETIT_022176mg	Aspartate–prephenate aminotransferase	1.64 *	0.85	0.88
SETIT_034777mg	Anthranilate synthase alpha subunit 1, chloroplatic-related	1.26 *	1.16 *	0.40
SETIT_010798mg	Anthranilate synthase	1.99 *	0.83	1.23 *
SETIT_036040mg	Anthranilate phosphoribosyltransferase	1.70 *	0.44	0.79
Glutamine family amino acid biosynthetic and metabolic processes				
SETIT_018043mg	Phosphoribosylanthranilate isomerase (PRAI)	1.88 *	1.07	1.69 *
SETIT_030453mg	SER/THR dehydratase, Tryp synthase	1.97 *	0.95	1.20 *
SETIT_013676mg	Tryptophan synthase beta chain 1, chloroplatic-related	1.51 *	0.89	1.03 *
SETIT_009345mg	Phenylalanine ammonia-lyase	1.99 *	1.39 *	1.20
SETIT_009509mg	Phenylalanine ammonia-lyase	-0.64	-1.39 *	0.08
SETIT_016475mg	Phenylalanine ammonia-lyase	1.86 *	1.73 *	2.11 *
SETIT_016504mg	Phenylalanine ammonia-lyase	1.45 *	-1.08 *	1.25 *
SETIT_013348mg	Phenylalanine ammonia-lyase	1.10 *	-0.77	0.00
SETIT_016478mg	Phenylalanine ammonia-lyase	0.63	-0.29	1.30 *

^1^ Fold change of gene transcript levels in AM roots compared with mock-treated roots. *, adjusted *p-*value < 0.05

### Comparing the expression of genes involved in cell wall construction

In mycorrhizal Hanevalval and ISE36, we observed that the Biological Process terms “cell wall biogenesis” (GO:0042546; adjusted *p*-value: 1.29 × 10^− 6^ in Hanevalval and 0.0007 in ISE36) and “cell wall polysaccharide metabolism” (GO:0010383; adjusted *p*-value: 0.0005 in Hanevalval and 0.0024 in ISE36) were significantly enriched in downregulated DEGs. To compare the influence of AMS on genes involved in cell wall biosynthesis and organization in three lines, we first retrieved the transcript levels of genes involved in the biosynthesis of cell wall components. Cellulose synthase A family (CesAs) and COBRAs are known to participate in cellulose biosynthesis (Li et al. 2003; Roudier et al. 2002). Among 14 *CesA*s genes in the foxtail millet genome, seven and two genes were significantly downregulated by AMS in Hanevalval and ISE36, respectively, while most *CesA* gene were not affected in TT8 and one *CesA* gene was even highly upregulated by AMS. Similarly, three out of eight *COBRA* genes were repressed in the mycorrhizal roots of Hanevalval (Additional file 1: Fig. S8a), but these three genes were not affected in TT8. Xyloglucan, xylan and mannans are the most abundant hemicelluoses that tether cellulosic microfibrils in the cell wall (McCann and Knox 2018). Many xyloglucan endotransglycosylase genes that are involved in xyloglucan biosynthesis (Hrmova et al. 2022) were downregulated in AMF-colonized Hanevalval and ISE36, whereas only few genes were affected in TT8. For cell wall-modifying enzymes, several genes such as pectate lyase, pectinase, glycosyl transferase, and endo-β-1,4-glucanase were also downregulated in AMF-colonized Hanevalval and ISE36 (Additional file 1: Table S6). In addition to polysaccharides, cell wall components also comprise proteins, including expansins that determine cell wall loosening. Twelve and four expansin A and B genes, respectively, were repressed by AMS in Hanevalval. In TT8, four and two expansin A and B genes, respectively, were downregulated, while only two expansin A genes were significantly increased in mycorrhizal ISE36 roots (Additional file 1: Fig. S8b). Based on the expression profiles, we observed the significant repression of cell wall biosynthesis and organization in mycorrhizal Hanevalval roots compared with TT8 and ISE36.

**Fig. 5:**
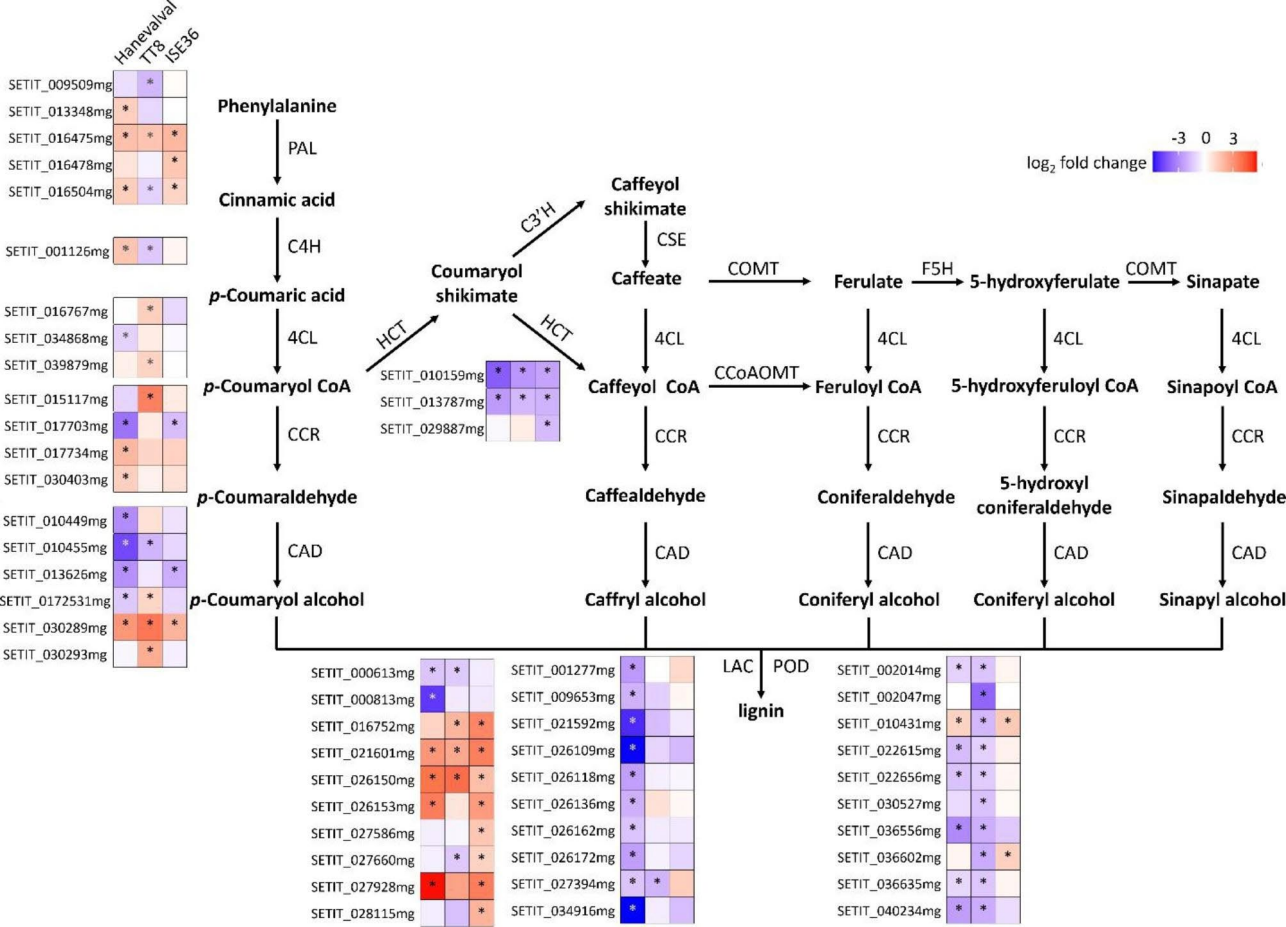
Differential expression of genes involved in monolignol biosynthesis and lignification. The scheme of lignen biosynthesis was modified from Xie et al. (2018). Red and purple indicate up- and downregulated genes, respectively, in mycorrhizal roots. *, adjusted *p*-value < 0.05. PAL, phenylalanine ammonia-lyase; C4H, cinnamate 4-hydroxylase; 4CL, 4-coumarate: CoA ligase; CCR, cinnamoyl-CoAreductase; CAD, cinnamyl alcohol dehydrogenase; HCT, quinateshikimate *p*-hydroxycinnamoyltransferase; C3′H, *p*-coumaroylshikimate 3′-hydroxylase; CSE, caffeoyl shikimate esterase; COMT, caffeic acid *O*-methyltransferase; F5H, ferulate 5-hydroxylase; CCoAOMT, caffeoyl-CoA *O*-methyltransferase; LAC, laccase; POD, peroxidase

Lignin is the important component of cell walls. The GO term “lignin metabolic process” (GO:0009808; adjusted *p*-value: 0.035 in Hanevalval and 0.0018 in ISE36) was significantly enriched in the down- and upregulated DEGs of Hanevalval and ISE36, respectively (Additional file: Figs. S6 and S7). We retrieved DEGs in this category and found that six cinnamyl alcohol dehydrogenase (*CAD*) genes were altered in different ways in the three lines (CAD enzyme functions in the core of the monolignol biosynthesis pathway (Tronchet et al. 2010). Among differentially expressed *CAD* genes, only one gene was commonly induced by AMF in all three lines. The responses of other five *CAD* genes to symbiosis varied between different lines (Fig. 5). We also examined the expression of genes encoding phenylalanine ammonia lyases (PALs) and 4-coumarate Co A ligases (4CLs), which function in the monolignol biosynthesis pathway, but only a few genes were altered (Fig. 5). Our results showed that the responses of monolignol biosynthesis genes to AMS were similar between Hanevalval and ISE36 but different in TT8.

After monolignols are formed, electron oxidation catalyzed by laccase (LAC) and peroxidase (POD) is important for lignification (Barcelo 1997; Freudenberg 1959; Takahama 1995). Here, we also observed the differential expression of *LAC*s and *POD*s in response to AMS. Laccases that are involved in monolignol polymerization belong to a class of multi-copper oxidases (Weng and Chapple 2010). Among 20 laccase genes altered in three lines, eight genes were upregulated in mycorrhizal ISE36 roots. In contrast, twelve and four members in this family were repressed and induced by AMS, respectively, in Hanevalval roots. In TT8, only three and two genes were up- and down-regulated by AMS, respectively. Of the ten *POD* genes retrieved from transcriptomic data, all of them were downregulated in TT8, only six genes were repressed in Hanevalval and two were induced in ISE36 (Fig. 5). In general, our results suggested that the lignification catalyzed by laccases could be enhanced in ISE36 and the lignification catalyzed by peroxidase was repressed in TT8. In Hanevalval, the downregulation of *LAC*s and *POD*s could have led to the reduction in lignification. However, more studies are required to understand how the complex transcriptional responses affect lignin formation in the roots.

## Discussion

In this study, we investigated the effects of AMS on different foxtail millet lines at the physiological and molecular levels. In general, inoculation with AMF increased the phosphate concentration in shoots but had no or negative effects on the growth of the crop (Fig. 1 and Additional file 1: Fig. S3). Studies of different crop species have shown that the mycorrhizal growth responses are affected by crop species, crop varieties, AMF species, soil fertility and growth conditions (Bernaola and Stout 2019; Campo et al. 2020; Eo and Eom 2009; Li et al. 2006, 2018; Wang et al. 2016, 2021). For example, in Li et al. (2018), the biomass and nutrient content in mycorrhizal wheat were significantly lower than mock-treated plants which might be attributed to high plant density and competition of nutrients between AMF and plants under low nutrient treatments. For supporting mycorrhization, up to 20% of fixed carbons in host plants is allocated to AMF (Parniske 2008). In our study, the plant height and biomass accumulaton of ISE36 and ISE42 were much higher than those of Hanevalval, which might positively correlate to the rate of nutrient depletion in the substrates. Thus, the negative effects of AMS on the biomass accumulation were more significant in ISE36 and ISE42 than in Hanevalval (Fig. 1 and Additional file 1: Fig. S3). Regarding crop yield, we observed no positive effects of AMS in TT8, while the panicle weight and thousand grain weight were significantly increased in ISE36, ISE42 and Hanevalval (Fig. 1). Herein, our data showed the impacts of the genetic variation of host plants on mycorrhizal growth effects.

The genome-wide transcriptome analysis of AMS-responsive genes has been carried out for many plant species, such as rice (Gutjahr et al. 2015), wheat (Li et al. 2018), *Medicago* (Liu et al. 2003) and tomato (Fiorilli et al. 2009). In our study, we investigated the transcriptome of foxtail millet in response to AMF colonization and the impacts of genetic variation on AMS responses. A total of 4,731, 4,206, and 2,170 DEGs were identified in Hanevalval, TT8, and ISE36, respectively. Although the AMF colonization efficiency was similar in all three lines, different numbers of DEGs suggested the effects of the variation of genetic background on AMS-mediated regulation. Genes conserved in AM host plant species are considered essential genes for symbiosis (Bravo et al. 2016; Delaux et al. 2014; Favre et al. 2014). The responses of most conserved genes to AMS were the same as previous reports (An et al. 2018; Bravo et al. 2016), and only a few genes behaved differently, supporting the conservation and importance of this group of genes in symbiosis. It is worth noting that the fold change varied by lines and that the induction levels were usually highest in TT8. We also observed the differential responses of some genes in three lines, e.g., the homolog of *CCD8b* was only induced in Hanevalval and ISE36 and the homolog of *CBF1* was upregulated in TT8 but downregulated in ISE36 (Additional file 1: Table S3). These results showed that different lines have significant impacts on the expression levels and responses of AMS-conserved genes which might lead to differential responses at physiological levels in host plants. The effects of genotype on the physiological and molecular responses to AMS have been reported previously for cassava and sorghum inoculated by one or two different AMF strains. In all 18 sorghum genotypes, the expression of *SbPT11* (an AMF-inducible *PHT1* gene) was significantly increased, but the magnitude was different between genotypes that do not always correlate with mycorrhization and growth responses (Watts-Williams et al. 2019). In cassava, around 72% of genes show genotype-dependent responses to AMF (Mateus et al. 2019). Considering the results of these studies, genotype variation has to be taken into account when interpreting symbiotic responses, as well as when applying AMF in fields and breeding highly compatible cultivars in the future.

Members of PHT1 family are key players for phosphate acquisition and redistribution in plants. It has been shown that in AM host plant species, the periarbuscular membrane-localized member that is specifically induced by symbiosis is responsible for utilizing fungal phosphate to maintain symbiotic relationship, e.g., MtPT4 in *Medicago* and OsPT11 in rice (Harrison et al. 2002; Javot et al. 2007; Yang et al. 2012). In addition, phylogenomic analysis has shown an AMF-specific phosphate transporter present only in monocots, though the role in AMS is still unclear (Yang et al. 2012). In Ceasar et al. (2014), *SiPHT1;8* and *SiPHT1;9* are both induced by AMS and belong to monocot-specific and general AMS-conserved *PHT1* genes, respectively. In contrast, we only observed the induction of *SiPHT1;9* in mycorrhizal roots, with the highest induction and lowest levels in TT8 and Hanevalval, respectively (Fig. 4a). We further confirmed that no *SiPHT1;8* transcript was amplified in mycorrhial roots by PCR (data not shown). One of reasons for this could be the differences in foxtail millet cultivars and AMF species. *Funneliformis mosseae* colonized millet cultivar Maxima was used in Ceasar et al. (2014), while in this study *C. etunicatum* was used as inoculants to examine AMS responses in foxtail millet. Grunwald et al. (2009) demonstrated the differential expression of *PHT1*s in *Medicago* roots colonized by three different AMF species. The induction of *MtPT1* by low phosphate was eliminated by *Rhizophagus irregularis* and *Gigaspora rosea* colonization but not by *F. mosseae*, implying the effects of AMF species on symbiotic responses. Using dual RNA-seq, Mateus et al. (2019) also revealed large amount of cassava genes affected by the interaction between plant and AMF genotypes. For example, a gene encoding a NAD(P)-BINDING ROSSMANN-FOLD SUPERFAMILY PROTEIN (Manes.01G053700) was significantly upregulated 5.5- and 2.7-folds by AMF isolate B1 and DAOM 197,198 colonization, respectively, in cultivar CM6438-14, but in cultivar CM4574-7, this gene was only induced less than 1-fold by B1 colonization and 1.2-fold by DAOM 197198. Further studies are required to understand the expression pattern and the molecular regulation of *SiPHT1;8* in response to colonization by different AMF species. In addition to AMS-conserved *PHT1*s, *SiPHT1;3*, *1;5* and *1;12* were significantly reduced in TT8; no other *PHT1* genes responded to AMS in ISE36; only *SiPHT1;3* and *SiPHT1;4* were down- and upregulated, respectively, in Hanevalval. According to gene expression data reported by Ceasar et al. (2014), *SiPHT1;3* and *SiPHT1;4* were induced by low phosphate in roots but showed no response to AMS, while *SiPHT1;5* was not detected under either phosphate-sufficient or -deficient conditions. Although the shoot phosphate concentrations in three lines were similar, the differential expression of *SiPHT1*s in TT8 compared with the other two lines suggested that the responses to internal phosphate levels are varied between lines or the fungal phosphate taken up by TT8 might be sufficient to reduce the transcript levels of phosphate starvation-responsive *PHT1*s.

It has been reported that AMF can increase nitrogen absorption and use-efficiency of host plants (Hodge and Storer 2015; Wu et al. 2020; Zhu et al. 2016). In this study, we did not observe an enhancement of biomass (Additional file 1: Fig. S3) or chlorophyll content (data not shown) in AMF-treated plants, but GO enrichment analysis showed that the Biological Function terms related to nitrogen transport and assimilation were mainly enriched in AMF-treated TT8 roots, not in other two lines (Additional file: Table S4 and Fig. S6). The upregulation of nitrate transporter and ammonia transporter genes has been reported in durum wheat and rice (Drechsler et al. 2018; Saia et al. 2015), and OsNPF4.5 was identified as a key player in the utilization of fungal N source (Wang et al. 2020). The ortholog of *OsNPF4.5* in foxtail millet (SETIT_004857mg) was also upregulated in all three lines studied here, but less nitrate transporters were affected in Hanevalval and ISE36 than in TT8 (Fig. 4b). A study of Populus nitrate transporter in colonized roots (Wu et al. 2020) suggested that the increase in nitrogen use-efficiency by AMF does not always correlate with the induction of *NRT* genes. Similarly, AMS-inducible ammonium transporters, including *MtAMT2;3* in *Medicago* and *SbAMT3;1* and *SbAMT4* in sorghum, were also identified (Breuillin-Sessoms et al. 2015; Koegel et al. 2013). Here, only one AMT ortholog (SETIT_004697mg) was commonly induced by AMS in all three lines, whereas the differential expression of other *AMT* genes was mainly observed in TT8 (Fig. 4b). Further research is required to decipher whether AMF affect nitrogen transport and acquisition in different ways at different levels in these lines.

In both Hanevalval and TT8, GO terms related to amino acid biosynthesis and metabolism were enriched in colonized roots (Additional file: Table S4 and Fig. S6). The metabolomic analysis also demonstrated an increase in the accumulation of amino acids in mycorrhizal roots, such as glutamic acid, aspartic acid, arginine, and cysteine (Cartabia et al. 2021; Dhawi et al. 2018; Lohse et al. 2005; Rivero et al. 2015), supporting the idea that AMF participates in reprogramming nitrogen assimilation. Glutamic acid is the precursor of several amino acids, such as glutamine and arginine. Although we did not analyze the contents of amino acids in roots, we coincidently found that genes involved in glutamine and arginine biosynthesis were upregulated in the AMF-colonized roots of Hanevalval and TT8 (Table 1), implying that the biosynthesis of amino acids derived from glutamic acid might be increased in AMF-treated roots.

Different from TT8, GO terms related to cell wall biogenesis, lignin and phenylpropanoid metabolic processes were the top ten enriched Biological Function terms in ISE36, while cell wall bionsynthesis and metabolism-associated terms were dominant in downregulated DEGs in Hanevalval (Additional File: Table S4 and Fig. S7), suggesting that the changes in the profiles of these secondary metabolites were the most significant impacts of AMS in these lines. Many genes involved in cellulose, hemicellulose and pectin biosynthesis in ISE36 and Hanevalval were downregulated by AMS (Additional fiel 1: Table S6 and Fig. S8). For genes involved in the monolignol biosynthesis pathway, the fold change by fungal colonization varied in the three lines. Interestingly, genes encoding laccase, which is involved in lignification, were upregulated in ISE36 but downregulated in Hanevalval. Peroxidases, another group of key enzymes for lignification, were downregulated in TT8 but not in ISE36 (Fig. 5). These results suggested that AMS could enhance lignification in ISE36 but repress in Hanevalval and TT8. It has been reported in many species that for fungal hyphae penetration and arbuscule formation, AMF colonization triggers cell wall reorganization in roots. In rice, the expression of cellulose and lignin biosynthesis genes was downregulated in mycorrhizal roots, leading to reductions in precursors of lignin (Gutjahr et al. 2015). Metabolomic analysis in tomato roots also showed the decrease in phenylalanine and tyrosine (the precursors of phenylpropanoid and lignin biosynthesis pathway) in mycorrhizal roots, but other intermediary compounds and monolignans were increased (Rivero et al. 2015). In contrast, the number of genes involved in the phenylpropanoid biosynthesis pathway was highly upregulated in mycorrhizal wheat roots (Li et al. 2018). According to transcriptomic analysis at the cellular level, a cellulose synthase gene was upregulated both in *Medicago* and *Lotus japonica* in arbuscule-containing cells (Guether et al. 2009; Liu et al. 2003). Significant increases in cellulose and hemicelluose in the mycorrhizal roots of switchgrass were found, but no difference in lignin content was observed (Basyal and Emery 2021). Based on these studies, AMF colonization reorganizes cell wall structure but the impacts on the biosynthesis and metabolism of cell wall components might be varied by cell types and host species. In our study, we further showed the impacts of genetic variation on the responses to AMS-triggered cell wall modification. Detailed studies are required to elucidate the cell wall profiles and cell wall strength/plasticity during AMS and the association of cell wall profiles with AMF colonization efficiency.

In conclusion, we investigated the impacts of AMS on the growth and yield production of different foxtail millet lines and performed comprehensive transcriptomic analyses to evaluate AMF-mediated molecular regulation. Our findings revealed the significant impacts of genetic variation on the physiological and molecular responses of host plants to symbiosis which need to be considered when applying AMF to crop production in the future.

## Electronic supplementary material

Below is the link to the electronic supplementary material.

Supplementary Material 1

## Data Availability

The datasets generated during the current study are available in the National Center for Biotechnology Information (NCBI) Gene Expression Omnibus (GEO) database (https://www.ncbi.nlm.nih.gov/geo/) (accession number: GSE213843). The data analyzed are included in this article and its additional file.

## Abbreviations

4CL: 4-coumarate-CoA ligase
AMF: arbuscular mycorrhizal fungi
AMS: arbuscular mycorrhizal symbiosis
AMT: ammonium transporter
ANOVA: analysis of variance
C3’H: p -coumarate 3-hydroxylase
C4H: trans-cinnamate 4 monooxygenase;
CAD: cinnamyl alcohol dehydrogenase
CCoAOMT: caffeoyl CoA 3-O-methyltransferase
CCR: cinnamoyl-CoA reductase
*C.*: *etunicatum Claroideoglomus etunicatum*
COMT: caffeic acid O -methyltransferase
CSE: caffeoyl shikimate esterase
DEG: differentially expressed gene
F5H: ferulic acid 5-hydroxylase
FDR: false discovery rate
GO: gene ontology
HCT: shikimate O-hydroxycinnamoyltransferase
LAC: laccase
*M.*: *truncatula Medicago truncatula*
PAL: phenylalanine ammonia lyase
POD: peroxidase
PHT: phosphate tranporter
SE: standard error
TT8: Taitung 8
