# Supplementary material for: Variation of growth and transcriptome responses to arbuscular mycorrhizal symbiosis in different foxtail millet lines
Source: Bot Stud. 2023 Jun 16;64:16. doi: 10.1186/s40529-023-00391-y (PMC10275850; doi:10.1186/s40529-023-00391-y)
Supplement: Supplementary file 1 — Supplementary Material 1 [file 40529_2023_391_MOESM1_ESM.pdf]

Table S1 Summary of RNA sequencing result.

Sample	Raw Read	Clean Read	Q20（%）	Q30（%）	GC content（%）
mock_TT8_1	44568702	40248420	98.95	95.87	52.79
mock_TT8_2	45385152	40304166	98.93	95.84	53.10
mock_TT8_3	50912562	45065510	98.96	95.93	52.49
mock_TT8_4	45112194	40226358	98.93	95.84	53.45
AMF_TT8_1	46180146	41099164	98.98	95.98	53.00
AMF_TT8_2	51528746	45979014	98.90	95.75	52.73
AMF_TT8_3	52873542	47491624	99.00	95.99	53.21
AMF_TT8_4	51523084	45882848	98.98	95.99	53.15
mock_Hanevalval_1	40884334	35832732	98.98	95.99	52.60
mock_Hanevalval_2	51127626	45475050	98.88	95.71	53.36
mock_Hanevalval_3	50536104	45394142	98.90	95.72	52.85
mock_Hanevalval_4	47735894	42621466	98.97	95.93	53.32
AMF_Hanevalval_1	52961304	46403550	98.94	95.86	52.72
AMF_Hanevalval_2	51684616	46114986	98.97	95.94	52.74
AMF_Hanevalval_3	50869318	45928178	98.95	95.88	52.77
AMF_Hanevalval_4	51126200	45065294	98.95	95.90	52.53
mock_ISE36_1	55416614	47234324	98.94	95.86	52.96
mock_ISE36_2	57419516	51ISE36780	99.01	96.05	52.99
mock_ISE36_3	55669328	49208972	98.98	95.99	53.04
mock_ISE36_4	54805980	49530964	99.01	96.05	53.09
AMF_ISE36_1	54681158	47810202	98.83	95.55	52.69
AMF_ISE36_2	55915296	49199660	98.84	95.57	52.88
AMF_ISE36_3	61331608	55208702	98.82	95.52	52.98
AMF_ISE36_4	54160154	47964184	98.80	95.45	52.93

Table S2 Summary of mapping result.

Sample	Total Reads	Total Mapped	Multiple Mapped	Uniquely Mapped
mock_TT8_1	40248420 (100 %)	38119827 (94.7 %)	2197582 (5.5 %)	35922245 (89.3 %)
mock_TT8_2	40304166 (100 %)	38444576 (95.4 %)	1783684 (4.4 %)	36660892 (91.0 %)
mock_TT8_3	45065510 (100 %)	42477209 (94.3 %)	2312641 (5.1 %)	40164568 (89.1 %)
mock_TT8_4	40226358 (100 %)	38368124 (95.4 %)	1617878 (4.0 %)	36750246 (91.4 %)
AMF_TT8_1	41099164 (100 %)	38041683 (92.6 %)	1460878 (3.6 %)	36580805 (89.0 %)
AMF_TT8_2	45979014 (100 %)	42696185 (92.9 %)	1462492 (3.2 %)	41233693 (89.7 %)
AMF_TT8_3	47491624 (100 %)	43977445 (92.6 %)	1497760 (3.2 %)	42479685 (89.5 %)
AMF_TT8_4	45882848 (100 %)	42661120 (93.0 %)	1514058 (3.3 %)	41147062 (89.7 %)
mock_Hanevalval_1	35832732 (100 %)	34118255 (95.2 %)	1275609 (3.6 %)	32842646 (91.7 %)
mock_Hanevalval_2	45475050 (100 %)	43365249 (95.4 %)	1741176 (3.8 %)	41624073 (91.5 %)
mock_Hanevalval_3	45394142 (100 %)	43153752 (95.1 %)	1807689 (4.0 %)	41346063 (91.1 %)
mock_Hanevalval_4	42621466 (100 %)	40695616 (95.5 %)	1484688 (3.5 %)	39210928 (92.0 %)
AMF_Hanevalval_1	46403550 (100 %)	41077600 (88.5 %)	1630073 (3.5 %)	39447527 (85.0 %)
AMF_Hanevalval_2	46114986 (100 %)	42613118 (92.4 %)	1648837 (3.9 %)	40964281 (88.8 %)
AMF_Hanevalval_3	45928178 (100 %)	42750868 (93.1 %)	1454701 (3.2 %)	41296167 (89.9 %)
AMF_Hanevalval_4	45065294 (100 %)	40835254 (90.6 %)	1875121 (4.2 %)	38960133 (86.5 %)
mock_ISE36_1	47234324 (100 %)	43810812 (92.8 %)	2038223 (4.3 %)	41772589 (88. 4 %)
mock_ISE36_2	51ISE36780 (100 %)	49197671 (96.1 %)	1713618 (3.4 %)	47484053 (92.7 %)
mock_ISE36_3	49208972 (100 %)	46887974 (95.3 %)	1549670 (3.2 %)	45338304 (92.1 %)
mock_ISE36_4	49530964 (100 %)	47330421 (95.6 %)	1402051 (2.8 %)	45928370 (92.7 %)
AMF_ISE36_1	47810202 (100 %)	44326652 (92.7 %)	1407065 (2.9 %)	42919587 (89.8 %)
AMF_ISE36_2	49199660 (100 %)	42849595 (87.1 %)	1549940 (3.2 %)	41299655 (83.9 %)
AMF_ISE36_3	55208702 (100 %)	52029557 (94.2 %)	1706686 (3.1 %)	50322871 (91.2 %)
AMF_ISE36_4	47964184 (100 %)	45087399 (94.0 %)	1681019 (3.5 %)	43406380 (90.5 %)

Table S3 The differential expression of AMS-conserved genes in three lines.

Gene ID	Annotation	Log$_2$ fold change *		
		Hanevalval	TT8	ISE36
SETIT_034161mg	Cation channel (Caster)	-0.09	0.01	0.04
SETIT_029334mg	Receptor-like kinase (DMI2)	**0.55**	0.24	0.28
SETIT_021787mg	Calcium/calmodulin-dependent protein kinase (DMI3)	-0.67	**0.75**	-0.66
SETIT_004288mg	Phosphate transporter (PHT1;9)	**4.00**	**7.08**	**6.58**
SETIT_008637mg	DNA-binding transcriptional activator (CYCLOPS)	**1.64**	**2.09**	0.88
SETIT_015555mg	ABCG half-ABC transporter (STR)	**1.29**	**3.63**	0.52
SETIT_003786mg	ABCG half-ABC transporter (STR2)	**2.44**	**5.55**	**2.43**
SETIT_012264mg	ABC transporter (ABCB12)	**2.36**	**5.86**	**1.80**
SETIT_015752mg	ABC transporter (ABCB2)	**-1.40**	1.06	1.04
SETIT_035002mg	MSP and ANK repeat-containing protein (VAPYRIN)	**1.14**	**1.81**	**1.48**
SETIT_035307mg	Glycerol-3-phosphate acyl transferase (RAM2)	**3.74**	**7.38**	**7.77**[#]
SETIT_027708mg	GRAS transcription factor (RAM1)	**4.20**	**7.78**	**6.99**[#]
SETIT_000959mg	GRAS family transcription factor (RAD1)	-0.72	**1.68**	-0.49
SETIT_021890mg	GRAS transcription factor (TF72)	**1.85**	**4.29**	-1.63
SETIT_003998mg	acyl-(acyl carrier protein) thioesterase (FatM)	**2.91**	**6.42**	**2.96**
SETIT_015320mg	protein binding protein (Exo71I)	**1.74**	**3.85**	0.45
SETIT_024947mg	ammonium transporter (AMT2:1)	**1.05**	**1.10**	0.84
SETIT_004697mg	ammonium transporter (AMT3:1)	1.16	**3.53**	**1.63**
SETIT_026160mg	oligopeptide transporter	-0.09	**-1.21**	0.13
SETIT_004857mg	nitrate transporter (ntr1)	**2.40**	**5.26**	**1.32**
SETIT_038848mg	AP2 domain protein (AP2c)	**4.23**	**3.89**	**3.28**
SETIT_032213mg	AP2/ERF domain transcription factor (AP2d)	ND	**2.67**	ND
SETIT_001909mg	AP2 domain transcription factor (AP2)	**3.75**	1.02	**4.21**
SETIT_022186mg	AP2 domain transcription factor (AP2)	**1.86**	**3.95**	0.18
SETIT_007857mg	AP2 domain transcription factor (AP2)	**4.39**	**7.06**	**7.30**[#]
SETIT_023636mg	CCAAT-binding transcription factor (CBF1)	-0.67	**1.94**	**-1.32**
SETIT_039424mg	Phytocyanin (BCP1)	1.36	ND	ND
SETIT_039030mg	plastocyanin-like domain protein	1.38	**7.98**	**5.81**[#]
SETIT_039259mg	plastocyanin-like domain protein	**4.35**[#]	**6.17**	ND
SETIT_020176mg	plastocyanin-like domain protein	1.56	**2.23**	1.43

Table S3 The differential expression of AMS-conserved genes in three lines (continued).

Gene ID	Annotation	Log_2 fold change *		
		**Hanevalval**	**TT8**	**ISE36**
SETIT_007296mg	plastocyanin-like domain protein	**-6.65**	-1.81	**-3.51**
SETIT_038020mg	plastocyanin-like domain protein	**1.42**	**1.84**	0.94
SETIT_011928mg	subtilisin inhibitor 1	**4.05**	**5.91**	**3.21**
SETIT_023913mg	subtilisin inhibitor	**2.55**	**6.27**	**2.40**
SETIT_019796mg	subtilisin inhibitor	**4.65**	**7.66**	**25.67**[#]
SETIT_033231mg	class III chitinase	**-4.89**	**-7.13**	**-3.93**
SETIT_021498mg	class III chitinase	**-2.71**	0.65	**-1.72**
SETIT_002402mg	class III chitinase	**4.56**	**7.22**	**2.03**
SETIT_002395mg	class III chitinase	**5.25**	**5.14**	ND
SETIT_004962mg	protein kinase	**2.80**	**3.74**	**1.78**
SETIT_028049mg	protein kinase	**3.16**	**5.06**	**4.71**[#]
SETIT_006522mg	protein kinase	**3.70**[b]	**5.89**	ND
SETIT_039206mg	late embryogenesis abundant (lea)	**5.43**	**7.90**[#]	**3.81**
SETIT_023002mg	late embryogenesis abundant (lea)	**-2.24**	-0.22	-0.44
SETIT_010999mg	late embryogenesis abundant (lea)	**-0.91**	-0.01	**-1.01**
SETIT_023221mg	late embryogenesis abundant (lea)	0.02	**-1.24**	-0.18
SETIT_032400mg	germin-like protein	**4.20**	**7.27**	**8.68**[#]
SETIT_033039mg	germin-like protein	ND	**7.77**	**3.94**[#]
SETIT_038965mg	Nod-factor receptor 5	**1.25**	0.72	0.40
SETIT_028233mg	heparan-alpha-glucosaminide N-acetyltransferase	**2.20**	**6.04**	0.01
SETIT_017532mg	Protein of unknown function	**-2.15**	-0.91	**-1.65**
SETIT_017497mg	GDSL-like lipase/acylhydrolase	**-1.47**	**-1.25**	-0.76
SETIT_032023mg	DnaJ domain protein	**0.86**	**1.72**	0.69
SETIT_032512mg	DnaJ domain protein	-0.01	**2.74**	-0.61

* The log_2 fold change labeled in bold means the adjusted P value < 0.05. Those labeled in red and green mean the genes significantly up- and down-regulated in response to AMS. ND means not detectable.

\# These genes were induced specifically by AMS. The normalized mean of read number in mock-treated samples was lower than 1 but higher in AMF-treated plants.

Table S4 Biological Process terms unique to single specific millet line.

Hanevalval		TT8		ISE36	
**Biological Process terms enriched in upregulated DEGs**					
GO:0006520	cellular amino acid metabolic process	GO:0015706	nitrate transport	GO:0009808	lignin metabolic process
GO:0009072	aromatic amino acid family metabolic process	GO:0015858	nucleoside transport	GO:0019748	secondary metabolic process
GO:1901605	alpha-amino acid metabolic process	GO:1901642	nucleoside transmembrane transport	GO:0140115	export across plasma membrane
GO:1901607	alpha-amino acid biosynthetic process	GO:0010167	response to nitrate	GO:1901361	organic cyclic compound catabolic process
GO:0009073	aromatic amino acid family biosynthetic process	GO:0071941	nitrogen cycle metabolic process	GO:0046271	phenylpropanoid catabolic process
GO:0009423	chorismate biosynthetic process	GO:0006820	anion transport	GO:0046274	lignin catabolic process
GO:0046417	chorismate metabolic process			GO:0019439	aromatic compound catabolic process
GO:0043650	dicarboxylic acid biosynthetic process			GO:0002239	response to oomycetes
GO:0006568	tryptophan metabolic process			GO:0006090	pyruvate metabolic process
GO:0006586	indolalkylamine metabolic process			GO:0017001	antibiotic catabolic process
GO:0042435	indole-containing compound biosynthetic process			GO:0009607	response to biotic stimulus
**Biological Process terms enriched in downregulated DEGs**					
GO:0009250	glucan biosynthetic process	GO:0009408	response to heat		
GO:0016051	carbohydrate biosynthetic process	GO:0009266	response to temperature stimulus		
GO:0071669	plant-type cell wall organization or biogenesis	GO:0030001	metal ion transport		
GO:0071554	cell wall organization or biogenesis	GO:0042542	response to hydrogen peroxide		
GO:0071555	cell wall organization	GO:0006812	cation transport		
GO:0005982	starch metabolic process	GO:0034605	cellular response to heat		
GO:0005977	glycogen metabolic process	GO:0006873	cellular ion homeostasis		
GO:0006112	energy reserve metabolic process	GO:0030003	cellular cation homeostasis		
GO:0045229	external encapsulating structure organization	GO:0030091	protein repair		
GO:0019252	starch biosynthetic process	GO:0055082	cellular chemical homeostasis		

Table S4 Biological Process terms unique to single specific millet line (continued).

Hanevalval		TT8		ISE36	
**Biological Process terms enriched in downregulated DEGs**					
GO:0009832	plant-type cell wall biogenesis	GO:0006826	iron ion transport		
GO:0051274	beta-glucan biosynthetic process				
GO:0009664	plant-type cell wall organization				
GO:0030244	cellulose biosynthetic process				
GO:0051273	beta-glucan metabolic process				
GO:0030243	cellulose metabolic process				
GO:0046351	disaccharide biosynthetic process				
GO:0007018	microtubule-based movement				
GO:0009312	oligosaccharide biosynthetic process				
GO:0009415	response to water				
GO:0006928	movement of cell or subcellular component				
GO:0005984	disaccharide metabolic process				
GO:0009414	response to water deprivation				
GO:0007017	microtubule-based process				
GO:0009311	oligosaccharide metabolic process				
GO:0005992	trehalose biosynthetic process				
GO:0005991	trehalose metabolic process				
GO:0009808	lignin metabolic process				
GO:0046271	phenylpropanoid catabolic process				
GO:0046274	lignin catabolic process				
GO:0009834	plant-type secondary cell wall biogenesis				
GO:0000281	mitotic cytokinesis				
GO:0061640	cytoskeleton-dependent cytokinesis				

Table S5 Molecular Function terms unique to single specific millet line.

**Molecular Function terms enriched in upregulated DEGs**					
**Hanevalval**		**TT8**		**ISE36**	
GO:0008483	transaminase activity	GO:0015112	nitrate transmembrane transporter activity	GO:0004175	endopeptidase activity
GO:0016769	transferase activity, transferring nitrogenous groups	GO:0016614	oxidoreductase activity, acting on CH-OH group of donors	GO:0052716	hydroquinone:oxygen oxidoreductase activity
GO:0017172	cysteine dioxygenase activity	GO:0030145	manganese ion binding	GO:0016682	oxidoreductase activity, acting on diphenols and related substances as donors, oxygen as acceptor
GO:0042562	hormone binding	GO:0005337	nucleoside transmembrane transporter activity	GO:0005507	copper ion binding
GO:0016829	lyase activity	GO:0022804	active transmembrane transporter activity	GO:0004190	aspartic-type endopeptidase activity
GO:0016702	oxidoreductase activity, acting on single donors with incorporation of molecular oxygen, incorporation of two atoms of oxygen	GO:0042626	ATPase activity, coupled to transmembrane movement of substances	GO:0070001	aspartic-type peptidase activity
GO:0045548	phenylalanine ammonia-lyase activity	GO:0043492	ATPase activity, coupled to movement of substances	GO:0016679	oxidoreductase activity, acting on diphenols and related substances as donors
		GO:0004568	chitinase activity	GO:0016837	carbon-oxygen lyase activity, acting on polysaccharides
		GO:0016616	oxidoreductase activity, acting on the CH-OH group of donors, NAD or NADP as acceptor	GO:0003680	AT DNA binding
		GO:0045735	nutrient reservoir activity	GO:0004601	peroxidase activity
		GO:0015399	primary active transmembrane transporter activity	GO:0016684	oxidoreductase activity, acting on peroxide as acceptor
		GO:0015405	P-P-bond-hydrolysis-driven transmembrane transporter activity	GO:0004197	cysteine-type endopeptidase activity
		GO:0004312	fatty acid synthase activity	GO:0004553	hydrolase activity, hydrolyzing O-glycosyl compounds
		GO:0015318	inorganic molecular entity transmembrane transporter activity	GO:0016798	hydrolase activity, acting on glycosyl bonds
		GO:0042910	xenobiotic transmembrane transporter activity	GO:0019200	carbohydrate kinase activity

Table S5 Molecular Function terms unique to single specific millet line (continued).

Molecular Function terms enriched in downregulated DEGs					
**Hanevalval**		**TT8**		**ISE36**	
GO:0015631	tubulin binding	GO:0015318	inorganic molecular entity transmembrane transporter activity		
GO:0008017	microtubule binding	GO:0015267	channel activity		
GO:0008092	cytoskeletal protein binding	GO:0022803	passive transmembrane transporter activity		
GO:0035251	UDP-glucosyltransferase activity	GO:0008324	cation transmembrane transporter activity		
GO:0008236	serine-type peptidase activity	GO:0015491	cation:cation antiporter activity		
GO:0017171	serine hydrolase activity	GO:0046873	metal ion transmembrane transporter activity		
GO:0008422	beta-glucosidase activity	GO:0022890	inorganic cation transmembrane transporter activity		
GO:0003777	microtubule motor activity	GO:0015318	inorganic molecular entity transmembrane transporter activity		
GO:0015926	glucosidase activity	GO:0015267	channel activity		
GO:0003774	motor activity	GO:0022803	passive transmembrane transporter activity		
GO:0004620	phospholipase activity	GO:0008324	cation transmembrane transporter activity		
GO:0004252	serine-type endopeptidase activity	GO:0015491	cation:cation antiporter activity		
GO:0016759	cellulose synthase activity	GO:0046873	metal ion transmembrane transporter activity		
GO:0016760	cellulose synthase (UDP-forming) activity	GO:0022890	inorganic cation transmembrane transporter activity		
GO:0052716	hydroquinone:oxygen oxidoreductase activity				
GO:0004805	trehalose-phosphatase activity				
GO:0016765	transferase activity, transferring alkyl or aryl (other than methyl) groups				
GO:0004373	glycogen (starch) synthase activity				
GO:1990939	ATP-dependent microtubule motor activity				
GO:0004175	endopeptidase activity				
GO:0043295	glutathione binding				
GO:1900750	oligopeptide binding				

Table S6 The differential expression of hemicellulose biosynthesis genes in three lines.

Gene ID	Annotation	Log$_2$ fold change *		
		Hanevalval	TT8	ISE36
SETIT_007018mg	Xyloglucan endotransglycosylase	-1.53	-0.01	-1.86
SETIT_010719mg	Xyloglucan endotransglycosylase	-2.43	-0.45	-1.20
SETIT_010796mg	Xyloglucan endotransglycosylase	-1.66	0.20	-1.77
SETIT_014194mg	Xyloglucan endotransglycosylase	-3.15	-0.52	-1.78
SETIT_014215mg	Xyloglucan endotransglycosylase	-2.57	-0.59	-1.77
SETIT_014232mg	Xyloglucan endotransglycosylase	-2.12	-0.89	-1.06
SETIT_014233mg	Xyloglucan endotransglycosylase	-3.59	-1.41	-0.15
SETIT_014242mg	Xyloglucan endotransglycosylase	-4.50	0.00	0.24
SETIT_014215mg	Xyloglucan endotransglycosylase	-2.57	-0.59	-1.77
SETIT_017966mg	Xyloglucan endotransglycosylase	-2.46	-0.02	-0.48
SETIT_006487mg	Xyloglucan endotransglucosylase	-2.28	-0.84	-1.66
SETIT_017659mg	Xyloglucan endotransglucosylase	-1.33	-0.12	-1.13
SETIT_030570mg	Xyloglucan endotransglucosylase	-1.50	-0.79	0.04
SETIT_026698mg	Xyloglucan endotransglucosylase	-2.91	-0.09	-1.43
SETIT_034503mg	Xyloglucan glycosyltransferase 12-related	-2.43	-0.69	-0.90
SETIT_013296mg	Xyloglucan 6-xylosyltransferase	-1.48	-0.85	0.45
SETIT_028282mg	Xyloglucan 6-xylosyltransferase	-1.13	-1.09	0.18
SETIT_035653mg	Xyloglucan 6-xylosyltransferase-related	-1.65	-0.12	-1.00
SETIT_036401mg	1,4-beta-D-xylan synthase	-1.57	-0.25	-1.05
SETIT_005721mg	1,4-beta-D-xylan synthase	-2.22	-0.19	-1.38
SETIT_013204mg	1,4-beta-D-xylan synthase	-3.11	0.36	-1.78
SETIT_028873mg	1,4-beta-D-xylan synthase	-1.49	-0.63	-0.03
SETIT_006115mg	Galactoside 2-alpha-L-fucosyltransferase	-2.32	-1.17	0.00
SETIT_019763mg	Galactoside 2-alpha-L-fucosyltransferase	-1.27	-0.67	-0.40
SETIT_001623mg	Xylogalacturonan xylosyltransferase	-1.84	-0.65	-1.10
SETIT_001622mg	Xylogalacturonan xylosyltransferase	-2.34	-0.57	-1.15
SETIT_016912mg	Glucomannan 4-beta-mannosyltransferase	-1.94	-0.75	-0.53
SETIT_034919mg	Glucomannan 4-beta-mannosyltransferase	-3.15	-1.62	-0.63

Table S6 The differential expression of hemicellulose biosynthesis genes in three lines (continued).

Gene ID	Annotation	Log₂ fold change *		
		Hanevalval	TT8	ISE36
SETIT_009553mg	Glycosyl transferase family 8	-1.54	-0.24	-1.17
SETIT_001402mg	GALACTURONOSYLTRANSFERASE 15-RELATED	-1.57	0.02	-0.27
SETIT_000786mg	Pectin methylesterase	-2.32	0.98	-0.73
SETIT_021901mg	Pectinase	-2.90	-1.07	-1.44
SETIT_032233mg	Pectinase		1.36	-1.73
SETIT_001461mg	Pectate lyase superfamily protein	-1.66	-0.52	-1.11
SETIT_032032mg	Pectate lyase superfamily protein	-1.18	-0.80	-0.58
SETIT_001489mg	Pectinacetylesterase	-1.13	-0.27	-0.26
SETIT_035783mg	Pectinacetylesterase	-1.61	-1.10	-0.98
SETIT_033872mg	Callose synthase 3	-1.57	-0.25	-0.79
SETIT_000021mg	UDP-glucose-1,3-beta-D-glucan glucosyltransferase	-1.31	-1.18	-0.68
SETIT_005663mg	UDP-glucose-1,3-beta-D-glucan glucosyltransferase	-1.07	-0.53	-0.64
SETIT_026351mg	O-hydroxycinnamoyltransferase	-1.05	-0.06	-0.89
SETIT_000629mg	α-galactosidase A	-2.10	-0.72	-0.99
SETIT_000604mg	Glycosyltransferase	-2.68	-0.68	-0.60
SETIT_027450mg	perplasmic β-glucosidase-realted	-1.86	-1.07	-0.94
SETIT_017772mg	Polysaccharide biosynthesis	-2.24	-2.14	-1.29
SETIT_012578mg	Fasciclin domain (Fasciclin)	-2.75	0.62	-2.29
SETIT_022977mg	Fasciclin domain (Fasciclin)	-1.41	0.13	-0.63
SETIT_002509mg	Fasciclin-like arabinogalactan protein 11	-4.01	-1.17	-1.03
SETIT_035913mg	Nucleotide-diphospho-sugar transferase	-1.36	-0.67	-1.13
SETIT_021103mg	UDP-glucose--glucose-phosphate glucosyltransferase	-1.26	0.74	-0.80
SETIT_028795mg	Kinesin-like protein-related	-1.94	-0.74	-0.95
SETIT_035254mg	Microtubule-associated protein 70-5	-1.44	-0.09	-0.07
SETIT_028842mg	D-alanine--D-alanine ligase	-1.05	1.02	-0.21
SETIT_035726mg	PMR5 N terminal Domain (PMR5N)	-3.88	-1.86	-0.67

* The log_2 fold change labeled in bold means the adjusted P value < 0.05. Those labeled in red and green mean the genes significantly up- and down-regulated in response to AMS. ND means not detectable.

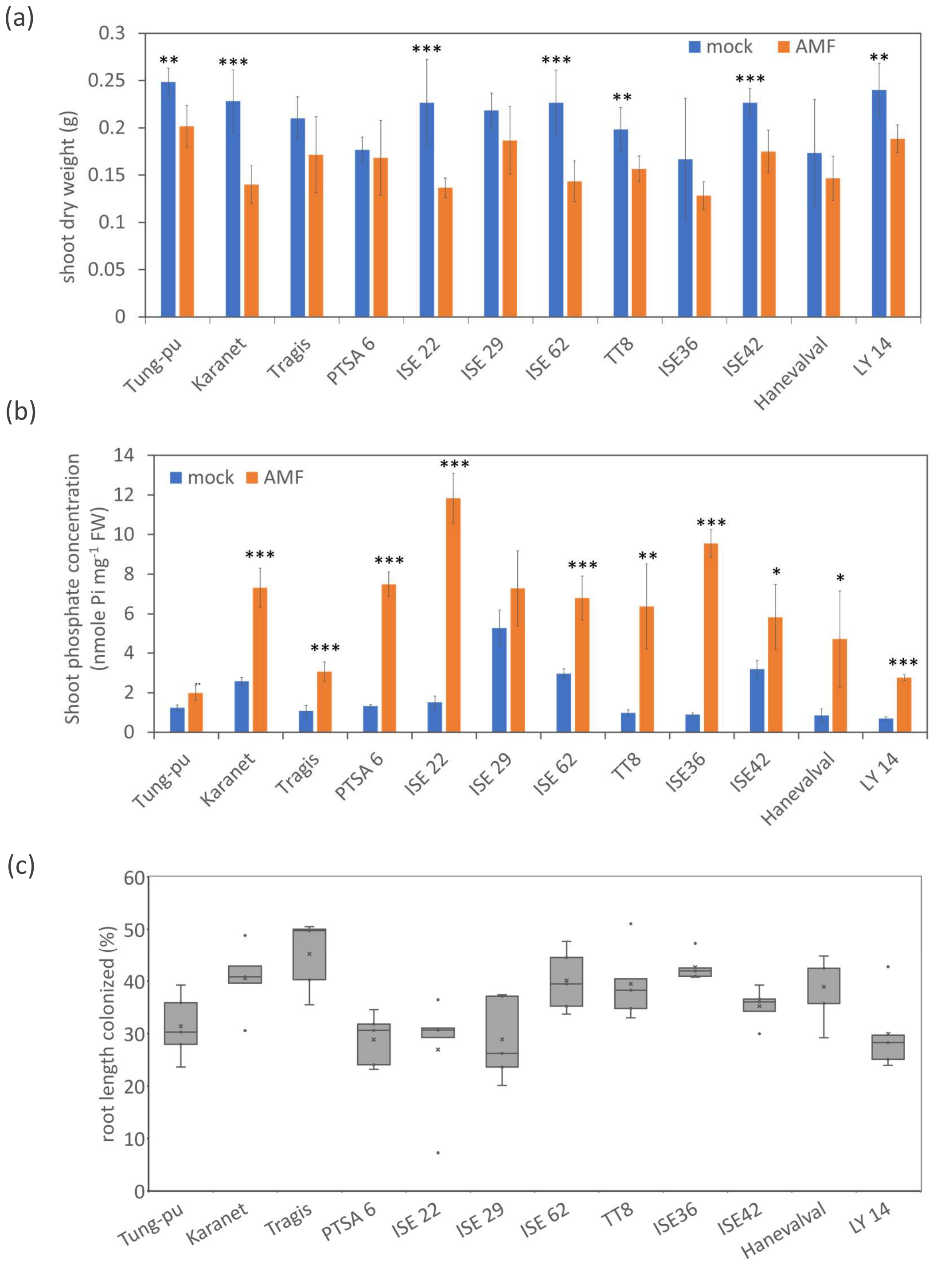

Fig. S1 The AMS phenotype of twelve different foxtail millet lines. a, Shoot dry weight; b, Shoot phosphate concentration; c, AMF colonization efficiency. The lines within the boxes mark the medians and the bottom and upper boundaries mark the 25th and 75th percentiles. n=5. Error bar = SE. The statistical significance between mock- and AMF-treated plants was evaluated by Student's t test. *, $p < 0.05$; **, $p < 0.01$; ***, $p < 0.001$.

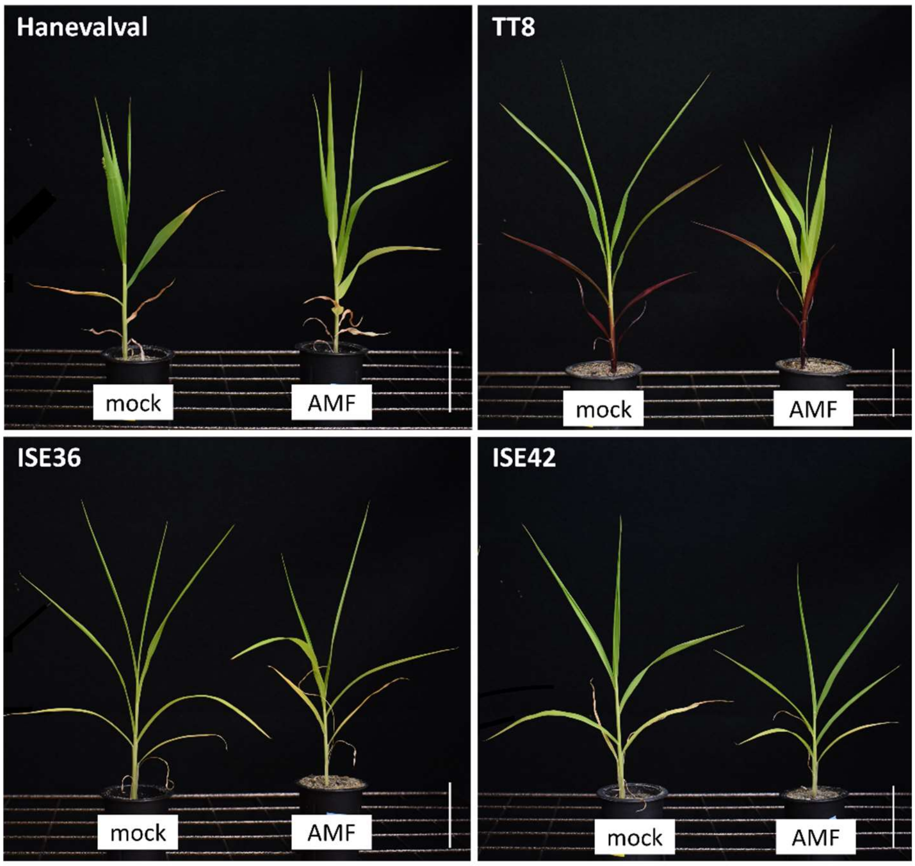

Fig. S2. The shoot morphology of mock- and AMF-treated foxtail millet lines. Bar = 5 cm.

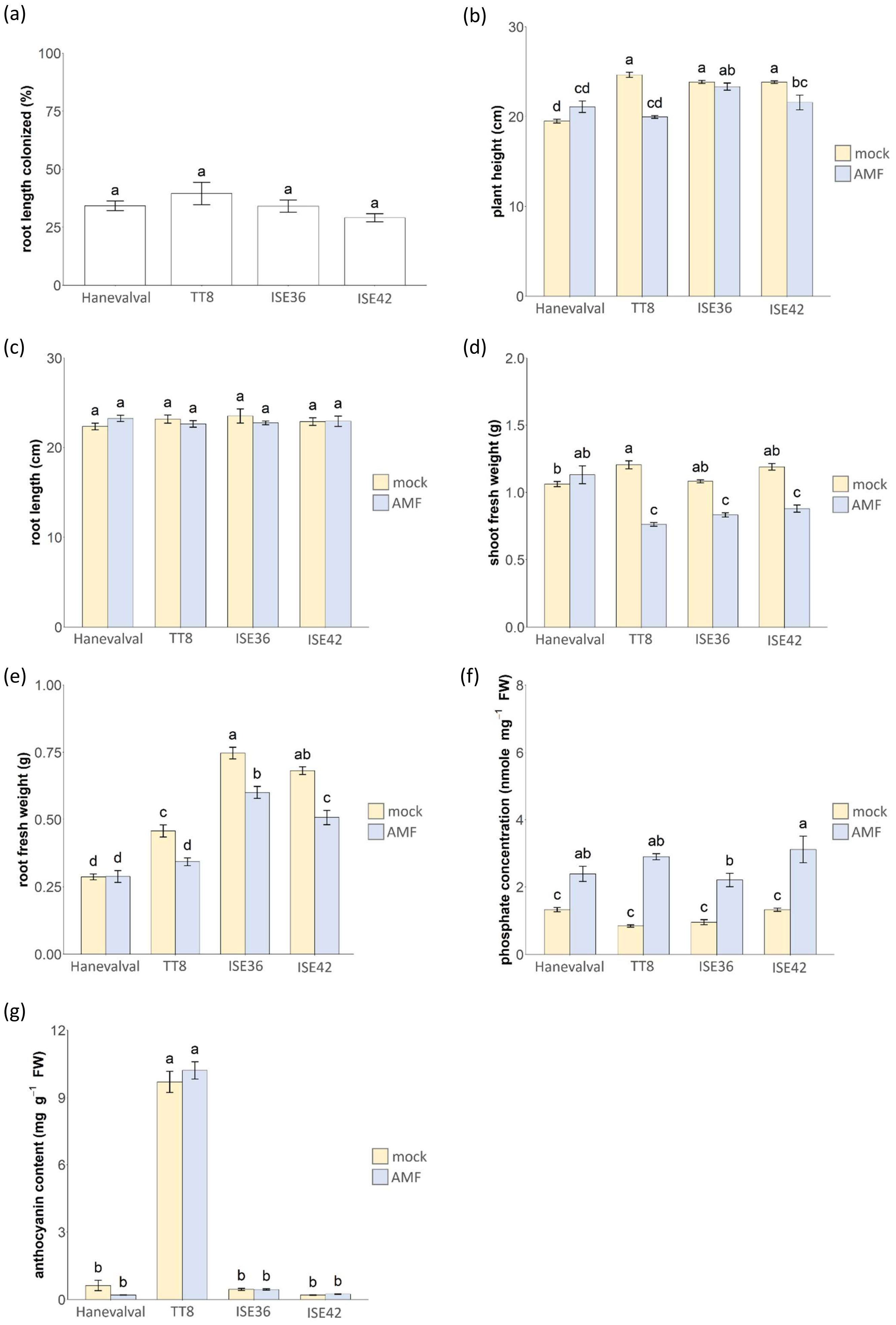
(a)
root length colonized (%)
100
75
50
25
0
a
a
a
a
Hanevalval
TT8
ISE36
ISE42
(b)
plant height (cm)
30
20
10
0
d
cd
a
cd
a
ab
a
bc
Hanevalval
TT8
ISE36
ISE42
mock
AMF
(c)
root length (cm)
30
20
10
0
a
a
a
a
a
a
a
a
Hanevalval
TT8
ISE36
ISE42
mock
AMF
(d)
shoot fresh weight (g)
2.0
1.5
1.0
0.5
0.0
b
ab
a
c
ab
c
ab
c
Hanevalval
TT8
ISE36
ISE42
mock
AMF
(e)
root fresh weight (g)
1.00
0.75
0.50
0.25
0.00
d
d
c
d
a
b
ab
c
Hanevalval
TT8
ISE36
ISE42
mock
AMF
(f)
phosphate concentration (nmole mg^-1 FW)
8
6
4
2
0
c
ab
c
ab
c
b
c
a
Hanevalval
TT8
ISE36
ISE42
mock
AMF
(g)
anthocyanin content (mg g^-1 FW)
12
9
6
3
0
b
b
a
a
b
b
b
b
Hanevalval
TT8
ISE36
ISE42
mock
AMF

Fig. S3 The AMS phenotypes of four different millet landraces. a, AMF colonization efficiency; b, Plant height; c, Root length; d, Shoot fresh weight; e, Root fresh weight; f, Shoot phosphate concentration; g, Anthocyanin content. n = 5. Values are mean ± SE. Data were analyzed with a two-way ANOVA ($p < 0.05$). Different characters over bars indicate significant differences.

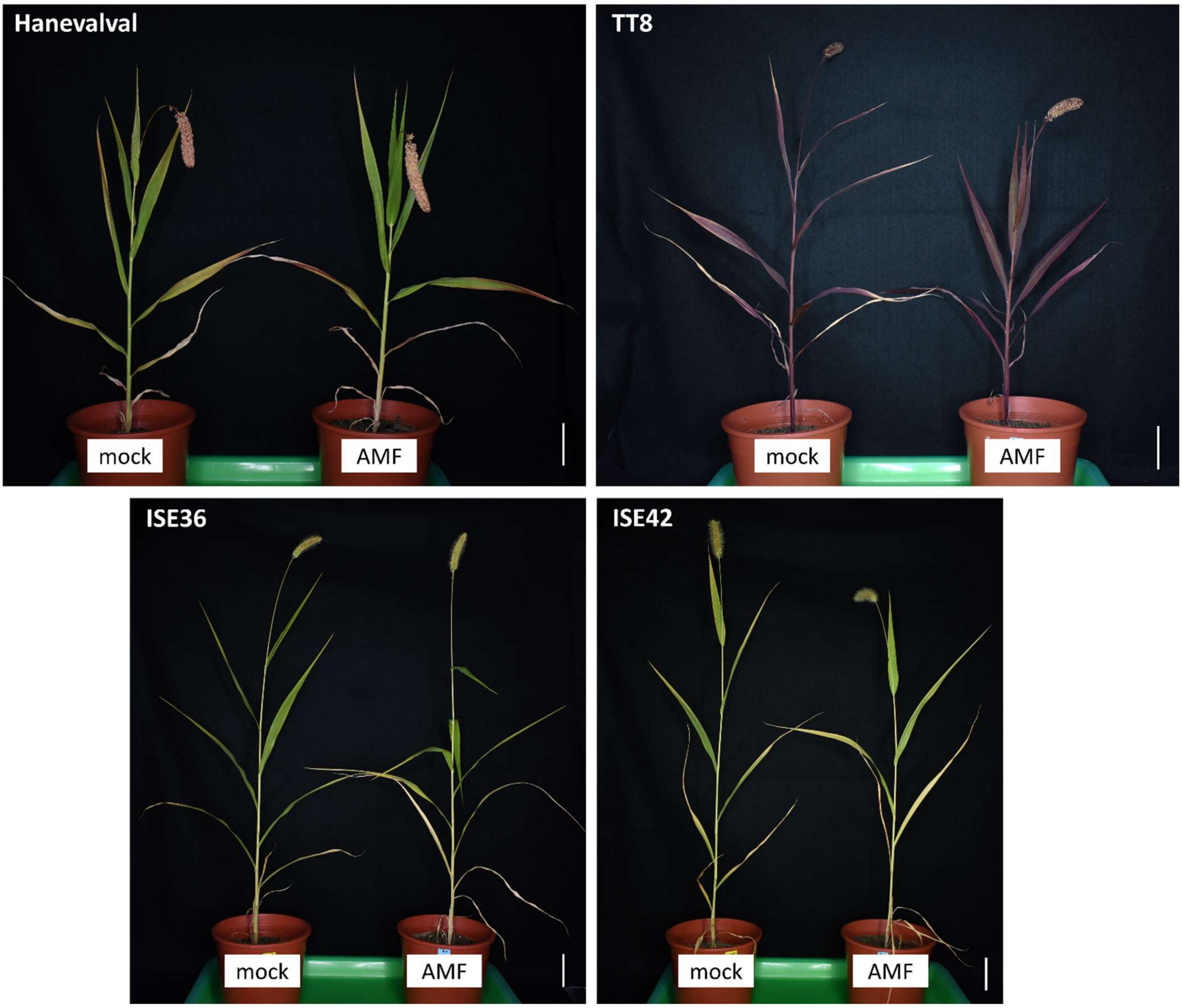

Fig. S4 The shoot morphology of mock- and AMF-treated foxtail millet lines during reproductive stage. Bar = 5cm.

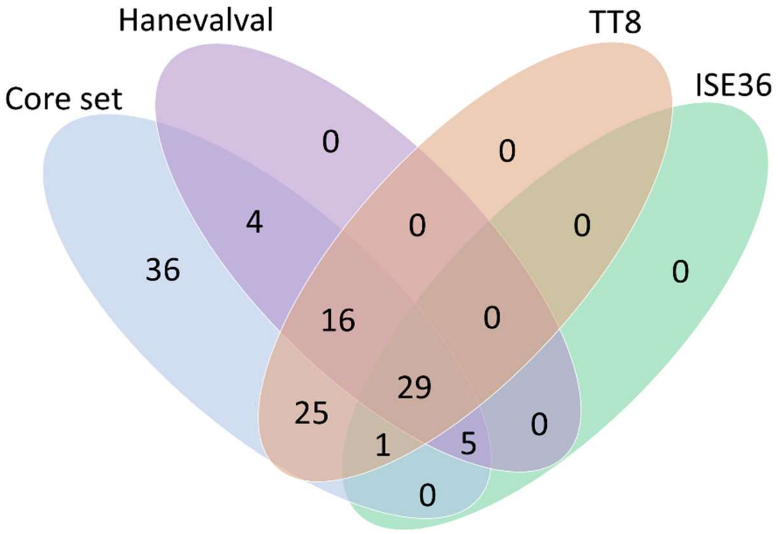

Fig. S5 The Venn diagram showing the number of DEGs in the core set of AMS-responsive genes in three lines.

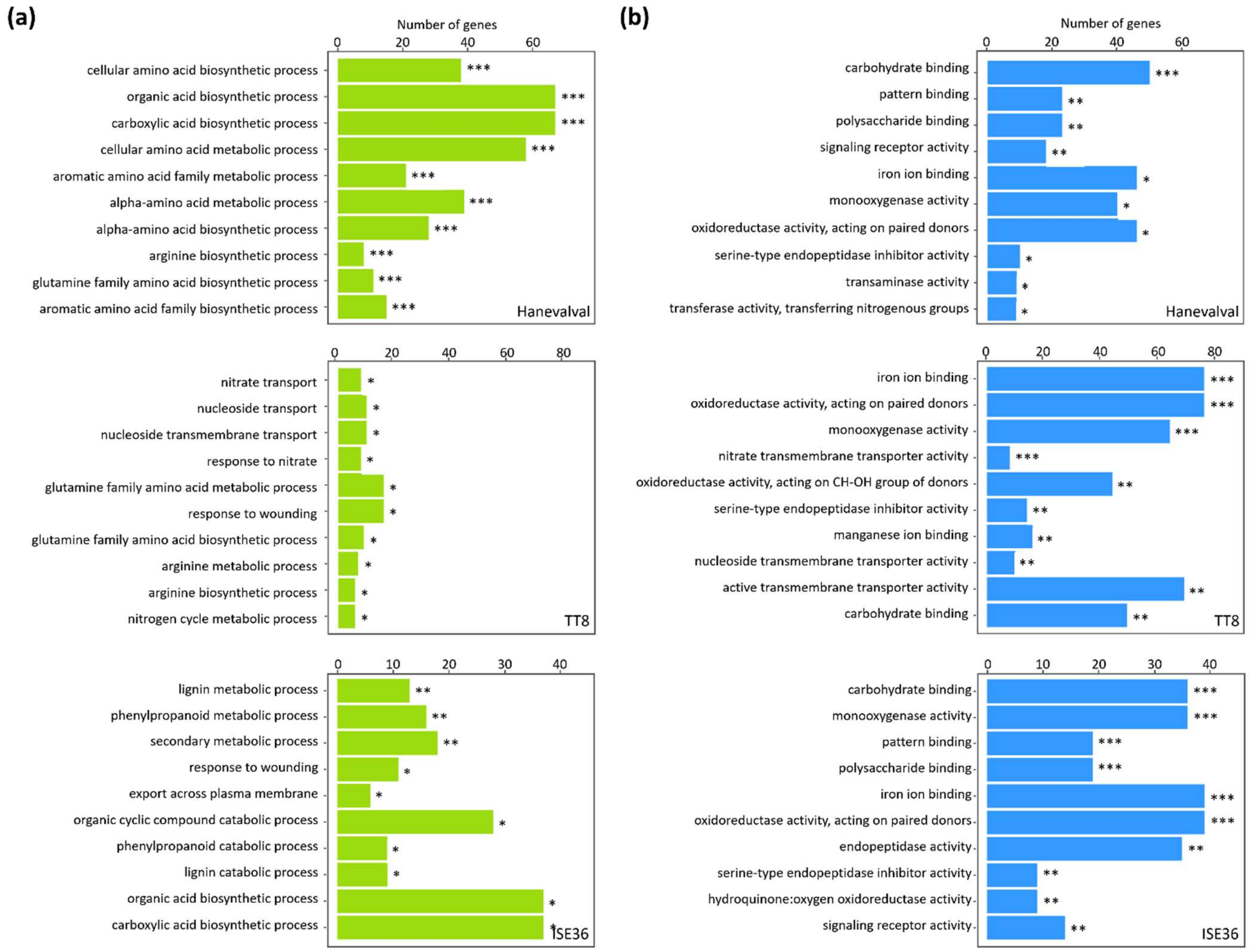

Fig. S6 Top ten GO terms enriched in upregulated gene profiles in three lines. a, Biological Process terms; b, Molecular Function terms. *, p-value < 0.05; **, p-value < 0.01; ***, p-value < 0.001.

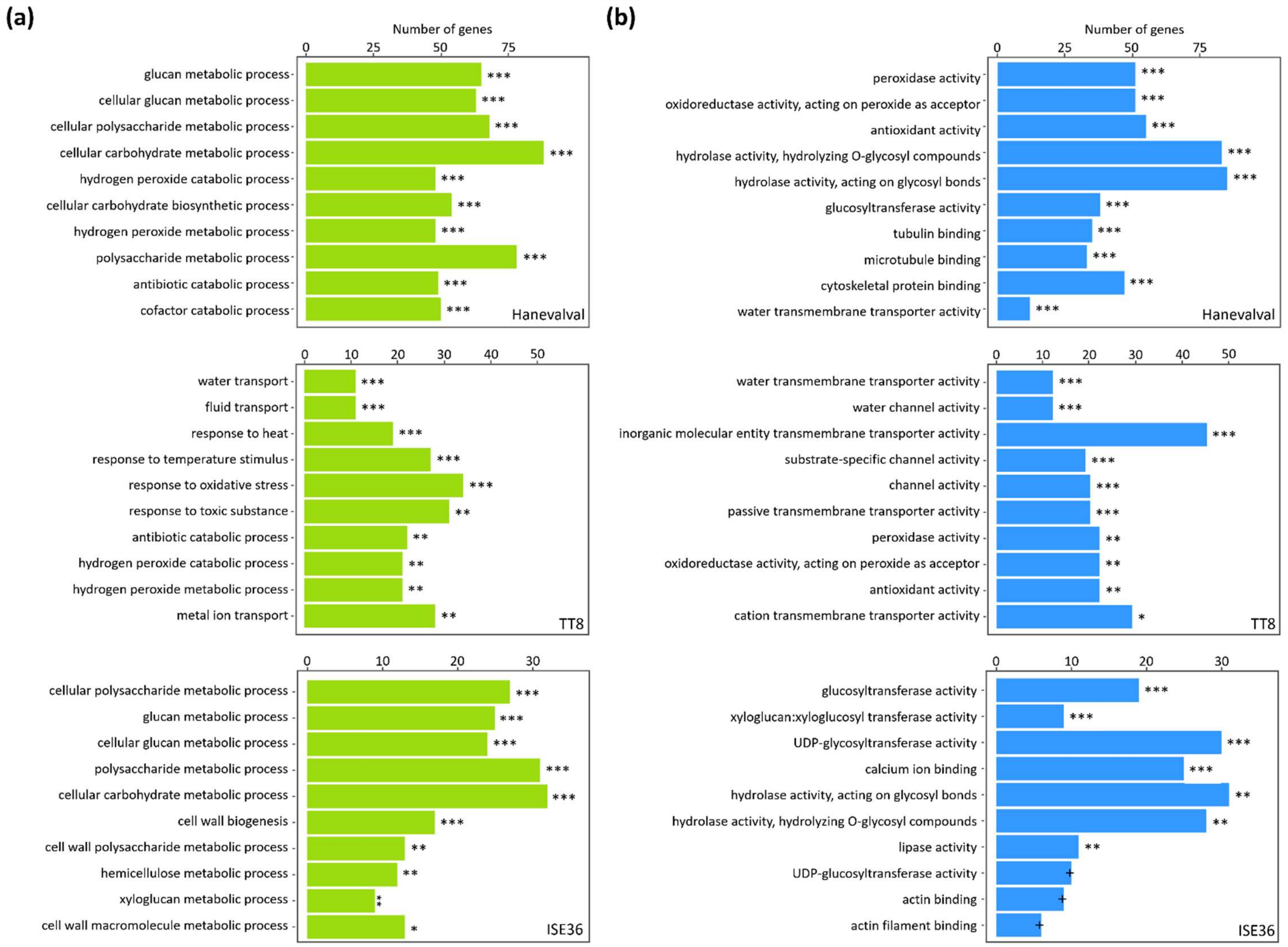

Fig. S7 Top ten GO terms enriched in downregulated gene profiles in the three lines. a, Biological Process terms; b, Molecular Function terms. +, p-value < 0.1; *, p-value < 0.05; **, p-value < 0.01; ***, p-value < 0.001.

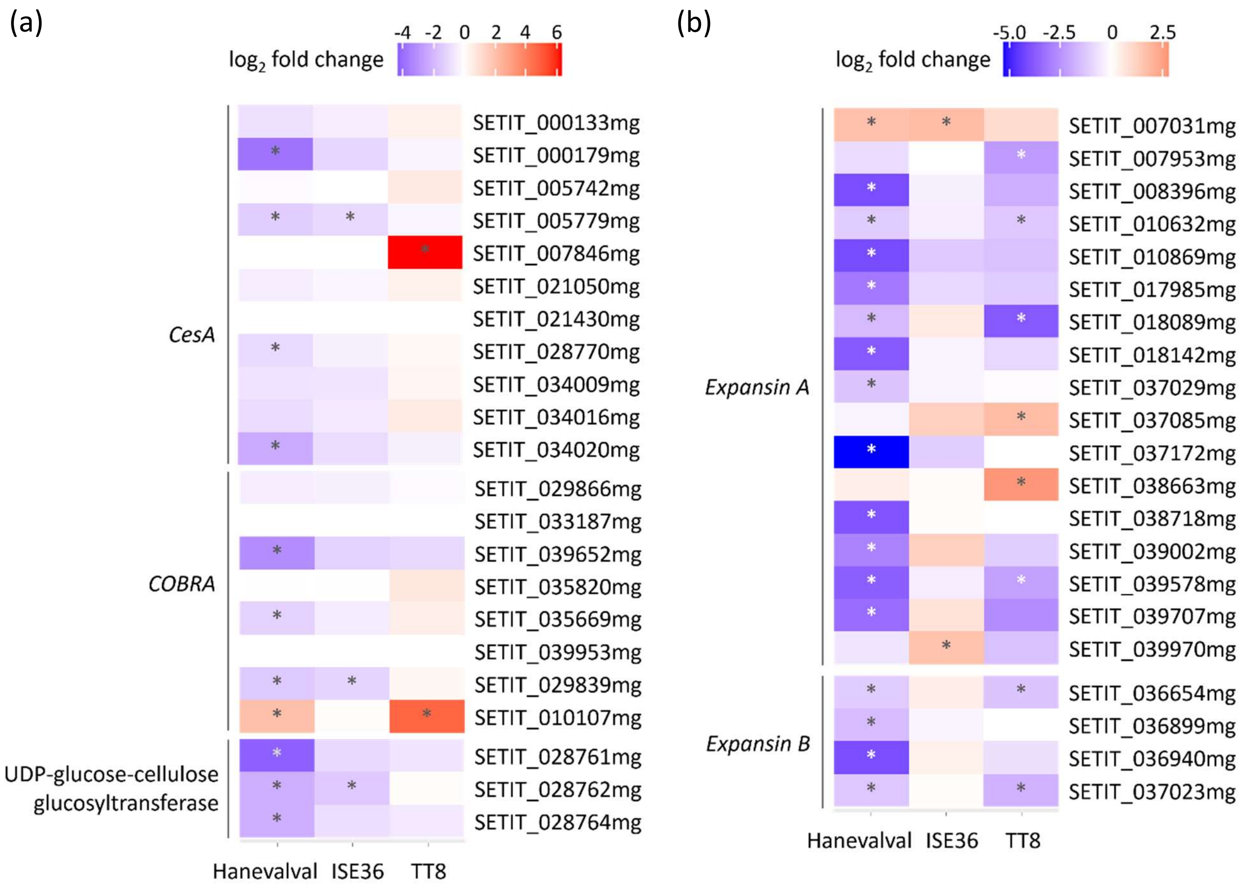

Fig. S8 Heatmap of a, *CesA* and *COBRA* genes; b, *Expansin A* and *B* genes that were significantly affected by AMS in all landraces. Red and purple indicate up- and downregulated genes, respectively, in AMS. *, adjusted *p*-value < 0.05.
